# The Well London program - a cluster randomized trial of community engagement for improving health behaviors and mental wellbeing: baseline survey results

**DOI:** 10.1186/1745-6215-13-105

**Published:** 2012-07-06

**Authors:** Gemma Phillips, Adrian Renton, Derek G Moore, Christian Bottomley, Elena Schmidt, Shahana Lais, Ge Yu, Martin Wall, Patrick Tobi, Caroline Frostick, Angela Clow, Karen Lock, Mark Petticrew, Richard Hayes

**Affiliations:** 1 Institute for Health and Human Development, University of East London, Water Lane, E15 4LZ, London, UK; 2 Tropical Epidemiology Group, Department of Infectious Disease Epidemiology, Faculty of Epidemiology and Population Health, London School of Hygiene and Tropical Medicine, Keppel Street, WC1E 7HT, London, UK; 3 Institute for Research on Child Development, University of East London, Water Lane, E15 4LZ, London, UK; 4 The Centre for Social and Health Outcomes Research and Evaluation, Level 7, 90 Symonds Street, Auckland, New Zealand; 5 Department of Psychology, University of Westminster, 309 Regent Street, London, W1B 2UW, UK; 6 Department of Health Services Research and Policy, London School of Hygiene and Tropical Medicine, 15-17 Tavistock Place, WC1H 9SH, London, UK; 7 Department of Social and Environmental Health Research, London School of Hygiene and Tropical Medicine, 15-17 Tavistock Place, WC1H 9SH, London, UK

**Keywords:** Cluster randomized trial, Community engagement, Health promotion, Physical activity, Healthy eating, Mental wellbeing, Social determinants

## Abstract

**Background:**

The Well London program used community engagement, complemented by changes to the physical and social neighborhood environment, to improve physical activity levels, healthy eating, and mental wellbeing in the most deprived communities in London. The effectiveness of Well London is being evaluated in a pair-matched cluster randomized trial (CRT). The baseline survey data are reported here.

**Methods:**

The CRT involved 20 matched pairs of intervention and control communities (defined as UK census lower super output areas (LSOAs); ranked in the 11% most deprived LSOAs in London by the English Indices of Multiple Deprivation) across 20 London boroughs. The primary trial outcomes, sociodemographic information, and environmental neighbourhood characteristics were assessed in three quantitative components within the Well London CRT at baseline: a cross-sectional, interviewer-administered adult household survey; a self-completed, school-based adolescent questionnaire; a fieldworker completed neighborhood environmental audit. Baseline data collection occurred in 2008. Physical activity, healthy eating, and mental wellbeing were assessed using standardized, validated questionnaire tools. Multiple imputation was used to account for missing data in the outcomes and other variables in the adult and adolescent surveys.

**Results:**

There were 4,107 adults and 1,214 adolescent respondents in the baseline surveys. The intervention and control areas were broadly comparable with respect to the primary outcomes and key sociodemographic characteristics. The environmental characteristics of the intervention and control neighborhoods were broadly similar. There was greater between-cluster variation in the primary outcomes in the adult population compared to the adolescent population. Levels of healthy eating, smoking, and self-reported anxiety/depression were similar in the Well London adult population and the national Health Survey for England. Levels of physical activity were higher in the Well London adult population but this is likely to be due to the different measurement tools used in the two surveys.

**Conclusions:**

Randomization of social interventions such as Well London is acceptable and feasible and in this study the intervention and control arms are well-balanced with respect to the primary outcomes and key sociodemographic characteristics. The matched design has improved the statistical efficiency of the study amongst adults but less so amongst adolescents. Follow-up data collection will be completed 2012.

**Trial registration:**

Current Controlled Trials ISRCTN68175121

## Background

Chronic diseases impose a large economic and social burden on health services, individuals, and communities in the UK [1-5]. It is estimated that physical inactivity in England and Wales carries direct and indirect costs in the region of £9 billion per year [6,7], which does not include the costs of overweight and obesity that could contribute a further £7 billion. The estimated costs of cardiovascular disease, to which physical inactivity and obesity contribute, is in excess of £30 billion [8]. Public health policies have repeatedly emphasized the need for preventive interventions that focus on increasing healthy eating and physical activity to reduce chronic disease incidence [9,10]. The complex interaction of individual, social, and environmental determinants of health behaviors is well-recognized [3,11-21], but few public health interventions that combine modification of the social and built environment with individual-level health promotion activities have been evaluated in the UK context [22].

In addition, there is a high burden of poor mental health in the UK: the point prevalence of depression, anxiety, and other non-psychotic mental health conditions amongst adults is estimated to be 18% [23]. The UK Foresight report on mental capital and wellbeing reported the annual costs of mental ill-health and reduced mental wellbeing in England to be approximately £77 billion, with more than half this cost being due to lost economic productivity [1]. Again, there is a need for interventions that act at both the individual and community levels to promote positive mental health and wellbeing [24]. Furthermore, the complex interactions of mental health with health behaviors and chronic diseases such as obesity and diabetes are well documented [1,3,25-31]. Therefore interventions that can address wellbeing in a holistic manner, seeking to improve mental health and wellbeing in addition to health behaviors, may have greater success in increasing physical activity and healthy eating.

The Marmot review of health inequalities in England is the most recent in a long series of reports highlighting that a large majority of health outcomes and health behaviors follow a strong social gradient in the UK, including physical inactivity, poor diet, and mental ill-health [32-36]. Significant spatial segregation by socioeconomic status in the UK has led to areas of concentrated deprivation, with clustering of poor health outcomes and a high prevalence of health-damaging behaviors [37-40]. There is now a plethora of studies seeking evidence about the social or physical characteristics of neighborhoods that may account for the persistence of poor health in these areas of high deprivation [41-46]. This social and geographical health inequity is further compounded by the lower success of traditional, individually-focused health promotion interventions amongst low-income and deprived groups [47-51].

The Well London program used a community engagement and co-production approach to design and deliver a suite of community-based projects with the aim of increasing physical activity, healthy eating, and mental health and wellbeing in 20 of the most deprived neighborhoods in London. The projects involved a mix of traditional health promotion interventions, community engagement activities, and changes to the physical neighborhood environment. The same framework for community engagement was used in all of the intervention sites, although the exact combination of projects delivered was tailored to local needs, in line with current theory on the design and evaluation of complex interventions [52,53]. The intervention program was funded by the UK Big Lottery Wellbeing Fund and was delivered by a partnership of community organizations and practitioners, led by the London Health Commission. Further information about the Well London intervention can be found on the Well London website [54] and in the published protocol [55]; the trial is funded by the Wellcome Trust.

The Well London intervention is being evaluated using a cluster randomized trial (CRT) [55], one of few such evaluations in the UK setting [56]. The CRT has four components: (1) a pre- and post-intervention cross-sectional household survey amongst adults resident in the intervention and control sites; (2) a pre- and post-intervention school-based survey amongst adolescents resident in the intervention and control sites; (3) a pre- and post-intervention structured neighborhood environmental audit in the intervention and control sites; and (4) a longitudinal qualitative component using participant observation and in-depth interviews in the intervention sites. The pre-intervention survey data collection was conducted during 2008; the post-intervention quantitative data collection is being conducted during 2011 and 2012.

The results of the baseline surveys and environmental audit are presented here, to assess the balance of key individual and area-level characteristics between the intervention and control sites. These include: the primary outcomes in adults and adolescents (levels of physical activity, healthy eating, and mental health and wellbeing); sociodemographic characteristics (age, gender, ethnicity, employment (adults only), level of education (adults only), duration of residence in the UK, duration of residence in the lower super output area (LSOA), family affluence (adolescents only)); and the neighborhood environment (residents’ perceptions of neighborhood safety, residents’ overall satisfaction with the neighborhood, walkability, cyclability, local amenities, local food stores, visual signs of incivilities). We present the matched coefficient of variation (K_m_) to demonstrate the between-cluster variance in the primary outcomes within pairs, which will be of use to other researchers designing studies to evaluate interventions targeting similar health and behavioral outcomes and present updated power calculations based on these empirical estimates of K_m_. We also present the unmatched coefficient of variation (K) to assess the impact of matching on the efficiency of the analysis. Finally, we briefly compare the health outcomes in the Well London adult survey population to the nationally representative Health Survey for England 2008.

## Methods

### The Well London cluster randomized trial

Full details of the CRT design are provided in the protocol [55], but are summarized briefly here. The unit of intervention delivery and analysis for the trial is the UK census LSOA; these are groupings of five to ten streets created for calculation of local area statistics in the UK census. Nationally, the mean number of residents in an LSOA is 1,500 people, with 800 to 1,000 residential addresses; the mean population, at the 2001 census, of the LSOAs included in the Well London CRT is 1,700 (range, 1,373 to 3,312).

The Well London intervention was delivered in 20 LSOAs with 20 matched control LSOAs. To ensure that the intervention was delivered in the most deprived LSOAs in London, and to ensure comparability between the intervention and control LSOAs the following selection process was used:

1. All 4,765 LSOAs in London were ranked by the English Indices of Multiple Deprivation (IMD) 2004 [57];

2. The 20 London boroughs containing the most deprived 11% of LSOAs were identified;

3. Within each of these 20 boroughs, the four most deprived LSOAs (based on the IMD) were identified;

4. Local authorities and health professionals were asked to select two LSOAs, which were not geographically contiguous, from the four identified in their borough;

5. Random allocation was used to assign one of the LSOAs to the intervention and the other became the control site.

### Study components

#### Household adult survey

Adults were interviewed in their homes by trained fieldworkers. Households were selected at random from the Post Office Address File for each of the 20 intervention and 20 control LSOAs, which contains a record for each Post Office delivery point. The addresses were assigned a number and a random number generator was used to select 150 addresses for the fieldworkers to visit. Each of the 150 addresses was visited on 5 separate days, at varying times of the day, before being classified as a non-responding address. At responding addresses, every eligible, consenting adult (aged 16 years and older) was interviewed independently. The target sample for each LSOA was 100 interviews. Further addresses were selected at random if 100 interviews had not been completed after visiting each of the 150 initial addresses five times. Where business addresses were selected and visited, they were removed from the sample and a replacement selected at random from the sampling frame. Written, informed consent was obtained from all participants.

The structured adult questionnaire contained validated measures of the three primary outcomes: healthy eating was assessed using a food frequency questionnaire adapted from the Health Survey for England [58]; physical activity was assessed using the International Physical Activity Questionnaire (IPAQ) [59]; positive mental wellbeing was assessed using the Snyder Hope Scale [60]; negative mental health was assessed using self-report consultation with a general practitioner for anxiety, depression, or a mental, nervous, or emotional problem and self-report feeling anxious or depressed (from the Euroqol 5D [61-63]). Additional file 1 shows the other domains that were collected, which included sociodemographic characteristics, and the source of the questionnaire items; the questionnaire is available from the authors on request.

The questionnaire was in paper format; fieldworkers read the questions to participants and recorded the responses on the questionnaire. Questionnaire responses were independently double-entered into a computerized database by two research assistants.

The response rate for the adult household survey was calculated at the household level as the percent of all households visited where at least one adult was interviewed. The individual-level adult response rate within households was calculated as the percent of all adults reported to be living in the household by survey respondents who were actually interviewed.

#### Adolescent school-based survey

The adolescent survey was administered to young people aged between 11 and 15 years who were resident in the intervention or control LSOAs. Recruitment and survey administration was coordinated through local secondary schools. Those schools situated near to the intervention or control LSOAs with 10 or more pupils resident in an LSOA were identified using data from the National Pupil Database, collated by the Department for Schools, Children and Families (now the Department for Education), and invited to join the study. All adolescents resident in the target LSOAs were invited to attend a 1 hour school timetable period, in a reserved classroom, to independently complete the paper questionnaire under the supervision of a fieldworker and a school teacher. Parents were contacted by letter prior to the questionnaire session to allow them to withdraw consent for their child to participate.

The structured adolescent questionnaire contained validated measures of the mental wellbeing and physical activity primary outcomes: negative mental health symptoms were measured using the Strengths and Difficulties Questionnaire (SDQ) [64]; positive mental wellbeing was measured using the Positive and Negative Affect Scale (PANAS) [65]; physical activity was measured using the Adolescent Physical Activity Questionnaire (PAQ-A) [66]. A food frequency questionnaire was included in the survey to measure overall dietary intake, with some additional general questions related to consumption of sweets and chocolate, sugar sweetened drinks, fried potato chips, fruit, breakfast, and water.

Additional file 1 shows the other domains of the questionnaire and the source of these items; the questionnaire is available from the authors on request. Questionnaire responses were independently double-entered into a computerized database by two research assistants.

#### Neighborhood environmental audit

The intervention and control LSOAs were visited by trained fieldworkers who completed a structured, paper-based audit tool covering the following domains: public green space; public amenities and services; cyclability; walkability; the food retail environment; the media environment (advertisements for food/drink or health promotion); and signs of social disorder and incivilities. Further details of the characteristics observed are provided in Additional file 1. The audit tool has been developed following a review of the literature of previous environmental audit instruments, and assessed with respect to its reliability and validity and will be published separately; a copy is available from the authors on request.

Two fieldworkers visited each site together, for safety reasons, but completed the audit form independently to allow cross-validation of the observations and agreed on the final data to be entered into a Microsoft Access database. Each LSOA was split into several segments (output areas) and the audit tool applied to each segment. Composite LSOA-level indicators were created from the multiple segments by summing or averaging the segment-level ratings, as appropriate.

### Primary outcomes

The primary outcomes to be assessed post-intervention in both adults and adolescents are levels of healthy physical activity, healthy eating, and mental health and wellbeing. Table 1 summarizes the indicators of these outcomes that were measured pre-intervention and which are used here to assess comparability of the intervention and control LSOAs at baseline.

**Table 1:** **Indicators of primary outcomes assessed pre-intervention**^**a**^

**Age group**	**Outcome**	**Indicator**	**Measurement tool**
Adults	Healthy eating*	Binary: consumption of five or more portions of fruit and vegetables per day (‘five-a-day’)	Food frequency questionnaire
Adults	Healthy physical activity*	Binary: doing five or more sessions of moderate intensity physical activity per week lasting at least 30 min (‘five-a-week’)	International Physical Activity Questionnaire
Adults	Mental wellbeing - positive	Continuous: Hope Scale score	Hope Scale
Adults	Mental wellbeing - negative	Binary: reports feeling anxious or depressed	EQ5D (1 item)
Adults	Mental wellbeing - negative	Binary: reports visiting GP for anxiety or depression or other emotional problem	Individual questionnaire item
Adolescents	Healthy eating - positive	Binary: frequent consumption of fruit	Individual questionnaire item
Adolescents	Healthy eating negative	Continuous: score summarizing frequency of consumption of chips, sweets or chocolate, and s ugar sweetened soft drinks^b^	Individual questionnaire items
Adolescents	Healthy physical activity*	Continuous: IPAQ score	Physical Activity Questionnaire for Adolescents
Adolescents	Mental health - negative*	Binary: score above threshold for normal mental health	Strengths and Difficulties Questionnaire
Adolescents	Mental health - positive wellbeing*	Continuous: positive affect score and negative affect score	Positive and negative affect scale

^a^Only those outcomes marked with an asterisk will be primary trial outcomes at follow-up. Mental wellbeing in adults will be assessed using the GHQ12 [67] and the Warwick-Edinburgh Mental Wellbeing Scale [68,69].

^b^Respondents completed a Likert scale to indicate the frequency of consuming these items; the overall unhealthy eating score was calculated as the mean response across the three items (scores: 1, ‘hardly ever’; 2, ‘once or twice a week’; 3, ‘3-4 times a week’; 4, ‘almost every day’; 5, ‘every day without exception’.

The analysis plan for the primary and secondary trial outcomes from the post-intervention surveys is provided in Additional file 2. The post-intervention survey is being conducted between March 2011 and March 2012 for the adult outcomes; the adolescent post-intervention survey will run to Autumn 2012. Two additional measures of mental wellbeing will be administered in the post-intervention adult household survey that are not reported here: the 12-item General Health Questionnaire (GHQ12) [67] that identifies negative mental health symptoms; and the Warwick Edinburgh Mental Wellbeing Scale, which is a UK-validated measure of positive mental wellbeing [68,69]. These will be used as the primary mental wellbeing outcomes for adults in the final trial analysis (see Additional file 2).

### Missing data in the Well London adult and adolescent surveys

Multiple imputation was used to account for missing data in the outcome indicators and key sociodemographic variables in the pre-intervention surveys, to increase power and reduce potential response bias [70-72]. Imputation was conducted separately for the adult and adolescent surveys; there were no missing data in the neighborhood environmental audit.

For outcomes comprising multiple separate questionnaire items (each of which can have missing data), each questionnaire item was imputed and the overall composite outcome score calculated from these imputed items. For example, the adolescent SDQ score has 25 component questions from which the overall score is calculated; missing responses for each of the 25 SDQ questions were imputed and then the overall score was calculated from these imputed values. For each questionnaire item within the composite scores, the imputation model included: the other individual questionnaire items from within the score; the overall calculated scores for the other outcomes; age (school year for adolescents); gender; ethnicity; duration of residence in the UK.

In addition, for adults only, the imputation model included: duration of residence in the LSOA; level of education attained; housing tenure; marital status; perceived ease of managing on the household income; smoking; level of self-reported alcohol consumption; self-reported primary health care consultation in past 12 months. For the adult healthy eating and physical activity outcomes the imputation model also included: self-reported chronic diseases (heart condition, diabetes); self-reported weight; for healthy eating only, the imputation model additionally included self-reported frequency of consumption of takeaway meals; for physical activity only the imputation model included self-reported respiratory problems and mobility problems. The adult mental wellbeing imputation model additionally included self-reported anxiety or depression and primary healthcare consultation in the past 12 months for these or other emotional/nervous or mental health problems. The imputation equations for the auxiliary variables (those used to impute the outcomes) included all other auxiliary variables and the overall outcome scores.

For adolescents only the imputation model additionally included the Family Affluence Scale [73].

The imputation model included indicator variables for LSOA, to account for clustering at the LSOA level.

The multiple imputation was conducted with the user-written ‘ice’ commands [74-80] in Stata v11.2 [81]. Twenty imputations were completed, with 20 cycles in each imputation. A complete case analysis was conducted to validate the analysis based on the multiply imputed data (major discrepancies between the MI analysis and complete case analysis could indicate an inappropriate imputation model). The complete case estimates of K, K_m_, and the ICC are based on cases providing responses for each outcome individually, rather than using one set of respondents who have data for every outcome considered; this is to increase the sample size available for the calculations. The results are reported in line with current recommendations on the use of multiple imputation in epidemiological analyses [82].

### Health Survey for England

The Health Survey for England dataset for 2008 was obtained from the UK Economic and Social Data Service online data-store. The Health Survey for England 2008 was used for comparisons of physical activity and healthy eating, smoking, and self-report feeling anxious or depressed at the time of interview (from the EQ5D). The sample sizes shown for the Health Survey for England are the effective sample sizes after accounting for design effect and survey weighting. Appropriate survey weights were used in regression models.

### Data analysis

All analyses were carried out using Stata v11.2 [81]. The response rate for the adult household survey was calculated at the household level as the percent of all households visited where at least one adult was interviewed. The individual-level adult response rate within those responding households was calculated as the percent of all adults reported to be living in the household by survey respondents who were actually interviewed.

Proportions and means, with confidence intervals based on robust standard errors to account for clustering at the LSOA level, are presented for each sociodemographic characteristic and health outcomes, separately for each trial arm. All summary statistics presented are based on the multiply imputed datasets. To allow comparisons to the national population, additional estimates of the Well London prevalences of meeting healthy eating and physical activity recommendations, daily smoking, and feeling anxious or depressed were produced by standardizing to the age-ethnicity distribution of the Health Survey for England population.

The data were used to estimate the matched and unmatched between-cluster coefficient of variation and the intra-cluster correlation coefficient (ICC) for each of the outcomes shown in Table 1.

The unmatched between-cluster coefficient of variation (K) is defined by:

(1) $$K=\sigma_{B}/m$$

Where $\sigma_{B}$ is the standard deviation of cluster (LSOA) means and *m* is the overall mean. For further detail on the method used to estimate K see Hayes and Moulton [83].

The matched between-cluster coefficient of variation (K_m_) is the average coefficient of variation within matched pairs (that is, within boroughs) and was estimated by (see Hayes and Moulton [83]):

(2) $$K_{m}=\sum K_{s}/S$$

where *S* is number of strata (boroughs), *K*_*s*_ is within stratum (borough) coefficient of variation and

(3) $$K_{s}={\hat{\sigma }}_{B_{s}}/m_{s}$$

where *m*_*s*_ is the overall mean in the *s*^th^ stratum (borough) and ${\hat{{\text{σ}}_{\text{B}}}}_{{}_{s}}$ is the estimated between-cluster variation in mean within strata (boroughs).

The intra-cluster correlation (ICC) compares the variability between clusters to the variability within and is defined as:

(4) $$\text{ICC}=\sigma_{B}{}^{2}/(\sigma_{B}{}^{2}+\sigma_{W}{}^{2})$$

It was estimated using within and between sum of squares obtained from a one-way analysis of variance, implemented in Stata, with the outcome as the dependent variable and LSOA as the independent variable.

### Ethical approval

Ethical approval for the study was received from the University of East London and London School of Hygiene and Tropical Medicine research ethics committees.

## Results

### Survey response

Adult survey: The household level response rate in the adult survey was 73% in the control LSOAs (standard deviation, 16; range, 41% to 99%) and 74% in the intervention LSOAs (standard deviation, 12; range, 41% to 94%). The overall contact rate across all intervention and control LSOAs was 85% and the active refusal rate was 13%. The mean individual-level response rate within responding households was 61% in both the intervention and control LSOAs. In total 4,107 adults were interviewed in the household survey, with a mean of 104 respondents per LSOA. The levels of missing data in the outcomes and key sociodemographic variables in the baseline survey are shown in Additional file 3.

Adolescent survey: There were 145 schools that had at least 10 pupils resident in one of the 20 intervention or 20 control LSOAs in the National Pupil Database. Sixty-eight schools were successfully recruited to take part in administration of the survey to pupils resident in the target LSOAs. The administrative records held by these schools indicated that approximately 57% of pupils (interviewed *n* = 1261) resident in the intervention and control LSOAs took part in the survey. Overall, those pupils represent 25% of adolescents recorded by the National Pupil Database (for England) as resident in the intervention or control LSOAs. Of the 1,261 pupils that completed the questionnaire, 14 were excluded from the analyses because they were in years 12 and 13 and a further 33 were excluded across three boroughs because sample size was too small to allow reliable imputation of the missing values in these LSOAs. In total, 1,214 adolescents were included in the analysis, with a mean of 47 respondents per LSOA. The levels of missing data in the outcomes and key sociodemographic variables in the baseline survey are shown in Additional file 3.

### Neighborhood audit

The mean number of segments assessed per area was five (minimum three, maximum eight) which was determined by the geography of the area; each street was treated as a segment. The majority of LSOAs had no shops selling fresh fruit and vegetables or a supermarket or general store, whereas the majority of LSOAs had at least one fast-food outlet (Table 2). All except one LSOA had moderate or high levels of physical signs of incivilities in at least one part of the LSOA, such as litter, graffiti, or broken windows in at least one segment surveyed within the LSOA.

**Table 2:** Adult health behaviours and health outcomes; prevalences and means across all respondents, adjusted for clustering within LSOAs; based on multiply imputed dataset

	**Control (*n*** **= 2,046)**	**Intervention (*n*** **= 2,061)**	**K across all**	**K**_**m**_**across all**	**ICC (ρ)**
	**(95% CI)**	**(95% CI)**	**LSOAs**	**LSOAs**	
*Trial outcomes*					
Healthy eating - meeting five-a-day %	38.3 (33.9, 42.7)	36.6 (33.1, 40.1)	0.20	0.14	0.02
Physical activity - meeting 5 x 30 min per week %	66.5 (61.2, 71.7)	63.4 (56.5, 70.3)	0.19	0.14	0.06
meeting 7 x 60 min per week %	25.5 (19.6, 31.3)	27.4 (19.2, 35.5)	0.50	0.42	0.10
Mental health - mean Hope Scale score^a^	4.6 (4.5, 4.7)	4.5 (4.4, 4.6)	0.04	0.03	0.05
Mental health - self-report feeling anxious or depressed %	18.7 (13.6, 23.8)	17.8 (13.6, 22.0)	0.50	0.30	0.05
Mental health - self-report visit to general practitioner for anxiety/depression %	15.6 (9.9, 21.3)	17.3 (11.3, 23.2)	0.71	0.23	0.10
*Other health outcomes*					
Smokes daily %	28.2 (23.4, 33.1)	27.4 (23.4, 31.4)	-	-	-
Self-report primary care consultation in					
past 12 months %					
No consultation	31.1 (22.5, 39.6)	29.5 (21.2, 37.8)	-	-	-
1 consultation	23.0 (19.9, 26.1)	22.2 (18.6, 25.9)	-	-	-
2 to 5 consultations	29.5 (24.7, 34.3)	29.3 (24.4, 34.1)	-	-	-
>5 consultations	16.4 (11.5, 21.4)	19.0 (14.1, 23.9)	-	-	-

^a^ Higher score indicates greater hopefulness; maximum score 6 (delivered using 6-point likert scale responses). CI, confidence interval; ICC, intra-cluster correlation coefficient; LSOA, lower super output area.

### Comparability of intervention and control groups

The intervention and control LSOAs were broadly comparable, particularly for the primary Well London CRT outcomes (Tables 3 and 4) in addition to the sociodemographic characteristics of adults (Table 2) and adolescents (Table 5) and the characteristics of the neighborhood environments in which they live (Table 6).The matched pair randomization based on the index of multiple deprivation has provided comparable intervention and control groups. However, the final trial analyses will still use adjustment for basic sociodemographic characteristics to check for any effects of minor imbalances between the groups, particularly the ethnic distribution of adolescent survey respondents (Table 5), and to increase the power to detect intervention effects (see Additional file 2).

**Table 3:** Adolescent health behaviours and health outcomes prevalences and means across all respondents, adjusted for clustering within LSOAs; based on multiply imputed dataset

	**Control**	**Intervention (n = 618)**	**K across**	**K**_**m**_** across**	**ICC (ρ)**
	**(n = 596)**	**(95% CI)**	**all**	**all**	
	**(95% CI)**		**LSOAs**	**LSOAs**	
*Trial outcomes*					
Diet					
Eat fruit daily or almost daily %	55.8 (51.7, 59.9)	57.5 (53.9, 61.0)	0	0.003	0
Unhealthy eating – mean score ^a^	3.0 (2.9 3.1)	2.9 (2.8, 3.1)	0.04	0.06	0.02
Physical activity – mean PAQ-A score ^b^	2.7 (2.6, 2.8)	2.8 (2.7, 2.9)	0.06	0.04	0.03
Mental health – mean PANAS positive score	33.0 (32.0, 34.0)	32.7 (31.9, 33.6)	0.004	0.03	0.002
– mean PANAS negative score	20.7 (19.9, 21.4)	19.9 (19.1, 20.6)	0.009	0.02	0.002
Mental health – mean SDQ score ^c^	13.1 (12.7, 13.4)	12.4 (12.0, 12.8)	0.0005	0.03	0
– normal SDQ score %	68.2 (65.3, 71.2)	72.5 (69.3, 75.7)	0	0.04	0

Abbreviations: CI, confidence interval; LSOA, lower super output area; ICC, intra-cluster correlation coefficient.

^a^ Possible range 1–5; higher score indicates more frequent consumption of unhealthy food items (chips, chocolate or sweets, and sugar sweetened beverages.

^b^ Range 1–5, 1 = very inactive, 5 = very active.

^c^ Borderline score = 16–19; abnormal score > =20.

**Table 4:** Sociodemographic characteristics of respondents in the adult household survey; based on multiply imputed dataset

	**Control (n = 2046)**	**Intervention (n = 2061)**
	**(95% CI)**	**(95% CI)**
Mean age in years	38.4 (36.6, 40.2)	38.0 (36.4, 39.5)
Gender % Female	52.7 (49.2, 56.2)	57.5 (54.6, 60.6)
Ethnicity %		
White British	28.9 (22.0, 35.7)	33.2 (25.5, 40.9)
White other	14.0 (9.8, 18.2)	12.6 (8.9, 14.2)
Black Caribbean	12.1 (8.2, 15.9)	11.4 (8.7, 14.2)
Black African	18.0 (12.2, 23.7)	15.6 (11.3, 19.8)
Indian/Pakistani/Bangladeshi	11.6 (4.7, 18.5)	9.3 (2.1, 16.5)
Other Asian	4.6 (2.1, 7.0)	4.3 (2.6, 6.1)
Mixed	4.5 (3.3, 5.6)	5.0 (3.2, 6.8)
Other	6.5 (4.1, 8.9)	8.6 (4.2, 12.9)
Marital status		
Never married	45.2 (41.6, 48.8)	43.9 (39.7, 48.2)
Married/cohabit	42.5 (38.0, 47.1))	41.9 (36.2, 47.7)
Separated	3.4 (2.3, 4.4)	3.1 (2.1, 4.2)
Divorced	5.2 (3.8, 6.6)	6.3 (4.6, 8.0)
Widowed	3.7 (2.6, 4.9)	4.7 (3.5, 5.9)
Mean duration of residence in the LSOA	16.8 (14.9, 18.7)	17.5 (15.7, 19.3)
Level of educational attainment		
No formal qualifications	8.8 (4.1, 13.5)	11.8 (7.5, 16.1)
GCSE or equivalent	32.2 (27.5, 37.0)	32.9 (27.4, 38.5)
A-level or equivalent	29.3 (26.0, 32.6)	27.8 (23.9, 31.5)
University degree	28.5 (23.2, 33.9)	26.7 (21.7, 31.8)
Other	1.1 (0.1, 2.2)	0.8 (0.1, 1.5)
Housing tenure		
Owner occupier	15.1 (11.8, 18.4)	12.3 (8.5, 16.1)
Rent/mortgage	1.2 (0.2, 2.2)	1.7 (0.5, 2.8)
Rent – social housing	51.5 (41.5, 61.5)	55.7 (45.5, 65.9)
Rent – private landlord	14.0 (9.5, 18.4)	12.0 (7.1, 17.0)
Other	18.3 (10.3, 26.3)	18.3 (11.1, 25.4)
Employed full or part time %	42.2 (37.1, 47.3)	42.8 (38.3, 47.3)
Ease of managing on household income		
Very easy	3.5 (2.2, 4.9)	2.9 (1.7, 4.0)
Fairly easy	18.3 (15.1, 21.5)	15.9 (11.5, 20.2)
Neither easy nor difficult	29.8 (22.9, 36.8)	28.0 (21.2, 34.8)
Fairly difficult	25.0 (19.9, 30.0)	28.2 (22.0, 34.4)
Very difficult	23.3 (16.9, 29.7)	25.0 (17.9, 32.2)

Abbreviations: CI, confidence interval.

**Table 5:** Sociodemographic characteristics of respondents in the adolescent school survey; based on multiply imputed dataset

	**Control (n = 596)**	**Intervention (n = 618)**
	**(95% CI)**	**(95% CI)**
School year %		
Year 7 (11–12 years)	26.5 (22.0, 31.1)	27.5 (23.0, 32.0)
Year 8 (12–13 years)	24.8 (21.4, 28.2)	24.6 (19.9, 29.3)
Year 9 (13–14 years)	19.1 (14.8, 23.5)	22.0 (16.5, 27.5)
Year 10 (14–15 years)	18.5 (15.4, 21.5)	16.8 (12.1, 21.5)
Year 11 (15–16 years)	11.1 (6.3, 15.9)	9.1 (4.3, 13.9)
Gender % Female	52.1 (47.2, 57.0)	49.2 (43.1, 55.3)
Ethnicity %		
White British	22.5 (10.8, 34.2)	22.4 (10.1, 34.7)
White other	4.1 (1.5, 6.7)	8.0 (3.6, 12.4)
Black Caribbean/other	8.6 (4.3, 12.9)	9.0 (4.9, 13.1)
Black African	19.7 (12.8, 26.5)	20.9 (12.5, 29.4)
Indian/Pakistani/Bangladeshi	23.7 (4.4, 43.0)	15.0 (6.5, 23.6)
Other Asian	3.9 (1.6, 6.1)	5.7 (1.7, 9.6)
Mixed	7.7 (4.7, 10.6)	10.5 (8.0, 13.1)
Other	9.8 (5.7, 14.0)	8.4 (5.4, 11.5)
Lived in UK all their life %	74.7 (65.6, 83.8)	71.5 (63.1, 80.0)
Family Affluence Scale Items %		
Family owns a vehicle	67.1 (62.0, 72.2)	68.2 (62.1, 74.3)
Own bedroom at home	49.6 (43.4, 55.7)	55.7 (48.9, 62.5)
Family owns a computer	86.9 (83.1, 90.7)	89.2 (85.8, 92.6)
Family holidays this year		
0	33.1 (28.5, 37.8)	30.4 (25.3, 35.5)
1	39.3 (35.4, 43.1)	34.9 (30.0, 40.0)
2	13.6 (9.6, 17.5)	16.7 (13.4, 20.0)
>2	14.0 (10.4, 17.5)	18.0 (14.9, 21.1)

Abbreviations: CI, confidence interval.

**Table 6:** **Environmental characteristics of the*Well London*CRT LSOAs**

	**Control (n = 20)**	**Intervention (n = 20)**
Area 1000 m^2^ - mean	187 (sd 177)	209 (sd 233)
Walkability score ^a^ - mean	5.1 (sd 2.7)	3.8 (sd 3.0)
Cyclability score ^b^ - mean	0.8 ( sd 0.8)	0.5 (sd 0.5)
Number of fast food outlets - median	0.5 (IQR 0, 4)	1 (IQR 0, 4.5)
Number of general grocery stores and supermarkets - median	0.5 (IQR 0, 1)	0 (IQR 0, 1.5)
Number of shops selling fruit and/or vegetables - median	0 (IQR 0, 0)	0 (IQR 0, 1.5)
Number of communal green spaces - median	9 (IQR 6.5, 19.5)	13.5 (IQR 9, 24)
Signs of home personalisation ^c^ - median	1.8 (IQR 1.5, 2.0)	1.6 (IQR 1.1, 2.2)
Neighbourhood watch signs/ prohibitive signs ^c^ - median	1.3 (IQR 1.0, 1.4)	1.4 (IQR 1.0, 1.3)
Incivilities ^d^ - median	1.1 (IQR 0.6, 1.6)	1.2 (IQR 0.6, 2.1)

^a^Walkability score calculated as the total number of paved pedestrian areas (not pavement), buffers between the pavement and road, signposts to aid pedestrians (for example, to landmarks such as station, library) and road-crossing aids, standardized by the total area of the LSOA in square meters.

^b^Cyclability score calculated as the total number of non-continuous and continuous cycle lanes and bike storage facilities, standardized by the total area of the LSOA in square meters.

^c^Mean score across all segments where: 1, none; 2, little; 3, moderate; 4, a lot.

^d^Composed of litter/broken glass, graffiti, broken/vandalized facilities, broken windows, security measures, unattended dogs, large dumped items in public space, dog foul, hyperdermic needles and syringes, alcohol bottles/cans, sex paraphernalia and condoms. Each item was rated: 1, none; 2, little; 3, moderate; 4, a lot. The composite score is the mean number of items across all segments assessed in an LSOA that were rated moderate or a lot.

IQR, interquartile range; sd, standard deviation.

### Between cluster variation in primary outcomes

The matched (K_m_) and unmatched (K) between-cluster coefficient of variation for each of the main trial outcomes is shown in Tables 3 and 4. There was generally less evidence of clustering by LSOA for adolescent health outcomes than for adults. Consequently, the matched design has reduced the between-cluster coefficient of variation substantially for the adult primary outcomes compared to an unmatched design with the same selected LSOAs (Table 3), but has had little effect on the coefficient of variation for the adolescent outcomes (Table 4). There was little difference in the estimates of K, K_m_, and the ICC between the multiple imputation and complete case analyses for the adult survey. There were some minor differences between the multiple imputation and complete case estimates of K and the ICC for the adolescent survey. There were minimal differences in the estimates of K_m_ for the adolescents, on which the power calculations were based. The complete case estimates of K, K_m_, and the ICC are shown in Additional file 4.

### Study power

The minimum detectable effect sizes for the primary outcomes, based on the baseline matched coefficients of variation, are shown in Tables 7 and 8. There are no widely accepted clinically relevant changes for the primary outcomes, but the study is sufficiently powered to detect the level of change predicted in the original sample size calculations completed at the beginning of the trial [55] and for many of the outcomes much smaller effect sizes will be detectable.

**Table 7:** Updated sample size calculations for the adult outcomes using the between-cluster coefficient of variation from the baseline survey (with missing responses multiply imputed)

**Outcome**	**Baseline prevalence or mean (across all intervention and control clusters)**	**Km**	**Minimum detectable effect size**	**Expected effect size in original study design**
*Adults*				
Healthy eating	37% of adults eating at least five portions of fruit and vegetables per day	0.14	22% increase in prevalence	50% increase in prevalence
Physical activity	60% of adults doing at least five sessions of 30 min of moderate intensity physical activity per week	0.14	19% increase in prevalence	70% increase in prevalence^b^
Mental health and wellbeing	18% of adults reporting feeling anxious or depressed	0.30	35% decrease in prevalence	-
	16% of adults reporting consulting their GP for emotional problems (anxiety and depression) in previous 12 months	0.23	41% decrease in prevalence	-
	Mean Hope Scale score = 4.6 (range, 1 to 6, higher score indicates better mental wellbeing)	0.03	Increase of 0.2 standard deviations	-

^a^Effect sizes for binary outcomes are relative increases in prevalence.

^a^Calculations are based on multiply imputed datasets; comparison to complete cases showed no substantial differences except in K_m_ for adolescent fruit consumption which decreased from 0.05 to 0.004.

^b^Based on an expected baseline prevalence of 18%.

**Table 8:** Updated sample size calculations for the adolescent outcomes using the between-cluster coefficient of variation from the baseline survey (with missing responses multiply imputed)

**Outcome**	**Baseline prevalence or mean (across all intervention and control clusters)**	**Km**	**Minimum detectable effect size**	**Expected effect size in original study design**
*Adolescents*				
Healthy eating	56% eat fruit daily or almost daily	0.003	17% increase in prevalence	30% increase in prevalence
Unhealthy eating score^b^	Mean unhealthy eating score = 3.0	0.06	Decrease of 0.26 standard deviations	
Physical activity^c^	Mean PAQ-A score = 2.7	0.04	Increase of 0.25 standard deviations	-
Mental health and wellbeing	Mean PANAS-positive score = 29.8 (ranges 11 = lowest positive affect, to 55 = highest positive affect)	0.03	Increase of 0.23 standard deviations	-
	Mean PANAS-negative score = 18.0 (ranges 11 = lowest negative affect, to 55 = highest negative affect)	0.02	Decrease of 0.21 standard deviations	
	Mean SDQ = 12.4 (range 0–15 = normal, 16-19 = borderline, 20- 40 abnormal)	0.03	Decrease of 0.22 standard deviations	30% increase achieving key thresholds for mental health
	74% Have normal SDQ scores	0.12	31% increase in prevalence	
	29% have borderline or abnormal SDQ scores	0.04	14% decrease in prevalence	

^a^Effect sizes for binary outcomes are relative increases in prevalence.

^a^Calculations are based on multiply imputed datasets; comparison to complete cases showed no substantial differences except in K_m_ for adolescent fruit consumption which decreased from 0.05 to 0.004.

^b^Possible range 1 to 5; higher score indicates more frequent consumption of unhealthy food items (chips, chocolate or sweets, and sugar sweetened beverages).

^c^Range 1 to 5; 1, very inactive; 5, very active.

### Comparison to national population

**Figure 1:**
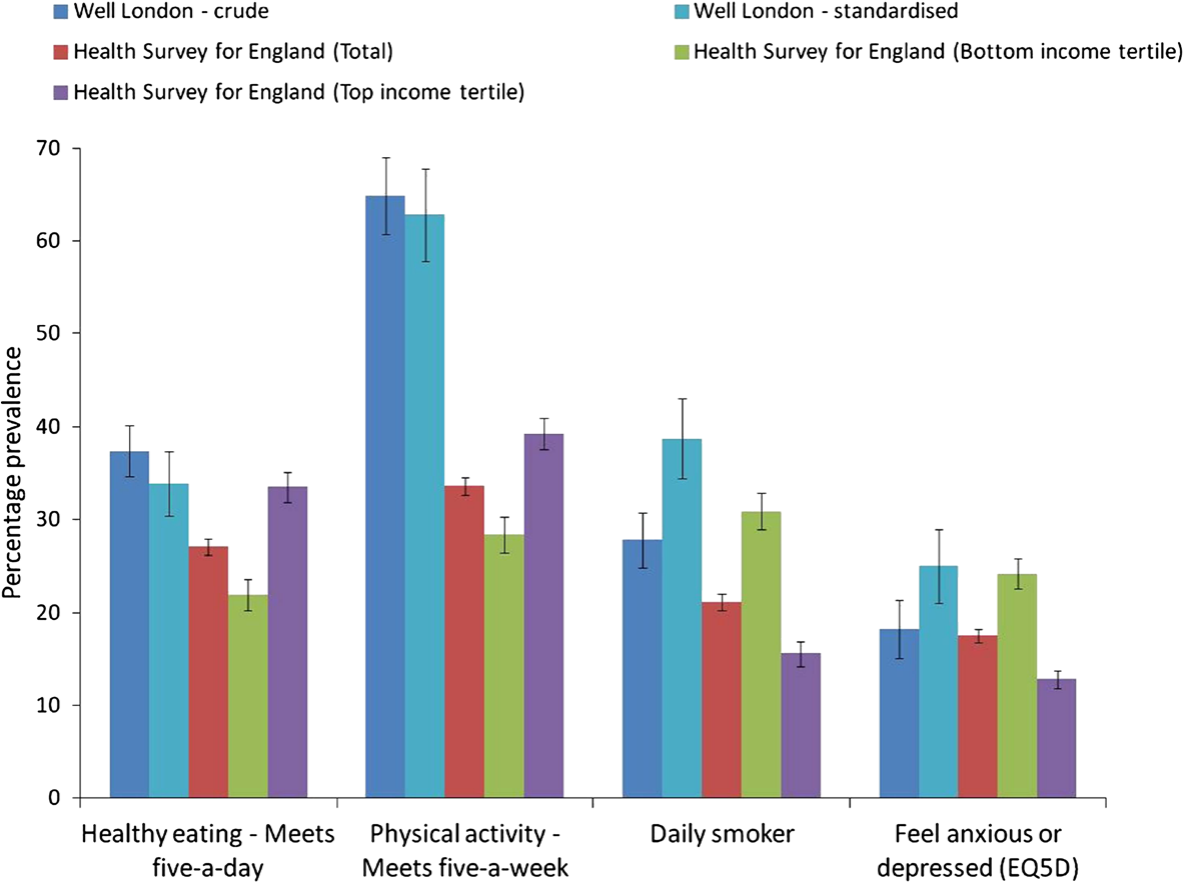
**Prevalence of health behaviors and outcomes in the Well Londonsurvey population and the national Health Survey for England.** Sample sizes: Well London = 4,107 (based on multiply imputed dataset); Health Survey for England total = 15,012; Health Survey for England lowest equivalized income tertile = 3,275; Health Survey for England highest equivalized income tertile = 4,327. Black bars represent 95% confidence intervals.

The crude prevalence of meeting the recommendation to consume at least five portions of fruit and vegetables daily was slightly higher in the Well London survey population (37%, 95% CI 35 to 40) compared to the national sample from the Health Survey for England (27%, 95% CI 26 to 28) (Figure 1). This was true for both the crude prevalences (Figure 1) and for ethnicity and age-stratified estimates (data not shown). The prevalence of meeting the recommendation to complete at least five sessions of 30 min of moderate intensity physical activity per week was substantially higher in the Well London population, but this may be attributable to the use of different data collection tools; the Health Survey for England used questionnaire items specifically designed and validated within the survey, whereas the IPAQ was used in the Well London survey. Age and ethnicity standardization of the Well London prevalences against the Health Survey for England population structure had little impact (Figure 1).

The prevalence of smoking was higher in the Well London population compared to the Health Survey for England. The prevalence of feeling anxious or depressed at the time of interview was similar in the two populations. After age and ethnicity standardization, the Well London prevalences of smoking and of anxiety/depression were higher than or similar to the prevalence in the lowest income tertile of the Health Survey for England population.

## Discussion

The Well London program is a unique, community-based intervention that uses a community engagement approach to deliver a program of projects to improve adult and adolescent health behaviors and mental wellbeing in the most deprived communities in London. Such a community-based approach is appropriately evaluated using a cluster randomized trial design. In order to account for geographic variation in social, economic, cultural, and environmental factors that may affect individual and community wellbeing, the intervention and control LSOAs were pair-matched on borough. The descriptive analysis presented in this paper shows that the matching and randomization processes have produced broadly comparable intervention and control populations in relation to sociodemographic and socioeconomic characteristics, the primary trial outcomes, and the local neighborhood environment. These deprived inner-London communities also seem to display levels of health and wellbeing in line with national trends.

In addition, we have presented estimates of the coefficient of between-cluster variation for the primary trial outcomes, which will be of use to other researchers studying similar outcomes in highly deprived populations in the UK, for the purpose of completing sample size and power calculations. Comparison of the matched and unmatched coefficients of variation indicates that matching within London borough was effective in reducing between-cluster differences in the primary outcomes for adults, but had little effect for the adolescents. The data presented here show that the trial arms are well-balanced for a number of key predictors of the trial outcomes. This is a major strength of the matched study design. Furthermore, in spite of the increased between-cluster variation in the adolescent survey with the matched design, the study is powered to detect small to moderate changes in both the adult and adolescent primary outcomes at follow-up.

Whilst the response rate at the household level in the adult survey was relatively high, with approximately three-quarters of households having at least one respondent, the response rate of eligible adults inside these households was estimated to be only 61%. This can be extrapolated to an estimated individual-level response rate of 50% for the whole LSOA across both responding and non-responding households. The overall response rate including non-responding households may be higher, if those households where no contact was made tend to have fewer residents than those where contact was made, which is a plausible mechanism of non-contact. However, the data still indicate that approximately half of all eligible adults in the survey sites actually participated in the survey. Whilst this is in line with other surveys conducted in deprived areas [84], such a low individual-level response rate raises concerns about selection bias. Lack of contact with any adult at an address and refusal after contact contributed similarly to the non-response. Therefore, in the follow-up survey, a number of measures have been taken to improve both contact rates and completed interviews. These include: (1) more stringent recruitment criteria for fieldworkers, requiring them to have substantial experience of interviewing or customer service/engagement; (2) improved fieldworker training and ongoing training and monitoring meetings throughout data collection; (3) use of computer-assisted personal interviewing, rather than paper questionnaires, allows real-time monitoring of fieldworker activity to increase efforts to make contact with selected addresses; and (4) conducting the survey in fewer areas at a given time to improve management of respondent recruitment by the coordinating team of researchers.

Unfortunately, the response rate in the school-based adolescent survey was lower than in the adult household survey. This was mainly due to difficulties in tracing adolescents from the target LSOAs into their schools because the National Pupil Database, which was used to develop the sampling frame, lags at least one school year behind. In addition, some schools were reluctant to take part in the surveys because they were already taking part in a number of local and national surveys and felt administratively over-burdened, in addition to concerns about student welfare and educational disruption and potential stigma attached to taking part in the Well London survey if fellow students knew that the intervention was targeted at particularly deprived areas. This had a substantial impact on the response rate in LSOAs where the majority of pupils attended a single school if that school did not participate in the survey. We would recommend better coordination of health and social surveys to reduce respondent burden and increase the efficiency of data collection. In addition, greater incentives are needed for schools to take part in area-based studies, where a few pupils across a number of year groups and schools are surveyed, because little useful information is generated about the student population at each school, which is a major compensation in school studies where the whole pupil body is surveyed. Similar difficulties have been encountered during the follow-up survey to date. Additional fieldworkers have been recruited to increase contact and liaison with schools and data collection has been extended to ensure that the required sample size is achieved.

There was a substantial amount of missing data in both the adult and adolescent surveys at baseline. Therefore we chose to use multiple imputation to reduce any potential bias associated with the non-response and improve the precision of the parameter estimates presented here. It is essential to explicitly account for the hierarchical structure of the dataset when carrying out the multiple imputation [85]. Ideally this would be achieved through the use of multilevel models in the multiple imputation, however there are no widely available computer packages for multilevel multiple imputation of the composite outcome scores and binary outcomes in the Well London baseline dataset. We therefore chose to use a fixed effect to represent the LSOA-level clustering in the multiple imputation, which was a pragmatic decision and an imperfect method. Whilst a recent simulation study has demonstrated that this approach may inflate the measures of within and between-cluster variation generated from the multiply imputed datasets [85], comparison of complete cases estimates of K, K_m_, and the ICC with the multiple imputation analyses indicate little impact of the imputation modeling approach on our particular dataset. Notably, the estimates of K_m_ used for the sample size calculations were particularly consistent between the complete case and multiple imputation analyses. Use of CAPI for the adult survey has greatly reduced the levels of missing data in the follow-up survey.

The Well London neighborhoods are amongst the 11% most deprived in London, and probably across the UK. However, the Well London survey population demonstrated similar levels of healthy eating to the national population in the Health Survey for England, even after age and ethnicity standardization. The age and ethnicity-standardized Well London prevalence of smoking was higher than in the bottom income tertile of the national population and the levels of self-report anxiety/depression were similar to this income group. The income measures were different in the two surveys; the Health Survey for England uses equivalized total annual household income whereas Well London survey respondents were asked to report their monthly ‘take home’ income (post-tax and social welfare payments). However, broadly translating between these income measures, almost two-thirds of the Well London population fall into the bottom income tertile bracket from the Health Survey for England. This may therefore indicate that in spite of a slightly higher average income, the Well London populations have poorer mental health and higher levels of smoking than people of similar income in the rest of England.

The levels of physical activity were substantially higher in the Well London population compared to the Health Survey for England. It is likely that some of this large difference is due to the use of different measurement tools; we used the IPAQ [59] in the Well London survey, whereas the Health Survey for England uses a specially developed questionnaire schedule. A recent systematic review indicated that the IPAQ may overestimate levels of moderate intensity physical activity [86], however the study quality was variable and only one study compared the IPAQ to the gold standard doubly-labeled water. The Health Survey for England physical activity module has not been validated against this gold standard. Whilst the measurement methods probably account for a substantial proportion of the difference in measured physical activity between the populations, it is possible that the Well London population is slightly more active because of differences in transport in inner-city areas compared to the whole of England. However, no transport modality data were collected in the Well London survey to examine this hypothesis.

Whilst the Well London program contains core health promotion elements, the use of community engagement is potentially transferable to many social interventions focusing on other topics such as environment and sustainability or anti-social behavior. There is ongoing debate about the use of randomized study designs for evaluation of complex social interventions [52,87-91]. The Well London CRT described here has demonstrated the feasibility and acceptability, to funders and stakeholders in statutory and third sector organizations, of using community randomization to deliver social programs, allowing rigorous evaluation of the outcomes. The flexibility of the funding source allowed the research team to have some control over where and when the intervention was delivered, which was key to the successful implementation of the randomization to conduct the CRT, as was the intensive and strategic development of partnerships between the Well London delivery organizations and local statutory organizations. Furthermore, the involvement of researchers from the beginning of the intervention development and funding bid was essential in building the evaluation design into program delivery. These are perhaps necessary conditions that are often not fulfilled by many government-funded programs, such as Sure Start in England, where political pressures take precedence over delivery planning that allows for a full CRT [53,91-93].

## Conclusions

The Well London CRT baseline survey has provided confirmation that the study has well-balanced intervention and control groups and is well-powered to detect moderate changes in the primary outcomes. This demonstrates the feasibility of using a randomized design for the evaluation of a complex, community-level intervention. The data have helped in the development of the analysis plan (provided in Additional file 2) and the follow-up surveys are now in progress, with completion of the adult outcome evaluation anticipated in March 2012.

## Abbreviations

CI, Confidence interval; CRT, Cluster randomized trial; GHQ12, 12 item General health questionnaire; IMD, Index of multiple deprivation; IPAQ, International physical activity questionnaire; IQR, Inter-quartile range; K, Between cluster coefficient of variation; Km, Pair-matched between cluster coefficient of variation; LSOA, Lower super output area; PANAS, Positive and negative affect scale; PAQ-A, Adolescent physical activity questionnaire; Sd, Standard deviation; SDQ, Strengths and difficulties questionnaire; UK, United Kingdom.

## Competing interests

The authors declare no competing interests.

## Authors’ contributions

AR and RH developed the CRCT design. AR, RH, MP, MW, and AC developed the overall CRCT protocol. MW and DM developed questionnaires. ES, SL, GY, PT, DM, and CF conducted data collection for the adult and adolescent surveys. MP and KL designed and conducted data collection for the neighborhood environmental audit. RH and CB provided statistical support. GP completed the analyses and drafted the manuscript. All authors contributed to revisions of this manuscript and approved the final version.

## Supplementary Material

Additional file 1  **[**[94-101]**].** Click here for file

Additional file 2 **Statistical Analysis Plan for the Well London Cluster Randomised Trial [**[7-30]**].** Click here for file

Additional file 3 Missing data in regression variables from the Well London adult baseline survey. Click here for file

Additional file 4 Adult health behaviors and health outcomes; prevalences and means across all respondents, adjusted for clustering within LSOAs; based on the complete case dataset. Click here for file
