# Supplementary material for: The Well London program - a cluster randomized trial of community engagement for improving health behaviors and mental wellbeing: baseline survey results
Source: Trials. 2012 Jul 6;13:105. doi: 10.1186/1745-6215-13-105 (PMC3441284; doi:10.1186/1745-6215-13-105)
Supplement: Additional file 1 — [[94-101]]. [file 1745-6215-13-105-S1.docx]

| **Data collection tool** | **Domains collected** | **Questions** |
| --- | --- | --- |
| Adult household survey | *Healthy physical activity* | International Physical Activity Questionnaire[[59](#_ENREF_1)] |
|  | *Healthy eating* | Food frequency questionnaire for fruit and vegetables adapted from Health Survey for England 2008[[94](#_ENREF_2)] |
|  | *Mental wellbeing* | The Hope Scale[[60](#_ENREF_3)] |
|  | *Social capital* | Questions from the Office for National Statistics Social Capital Harmonised Question Set[[95](#_ENREF_4), [96](#_ENREF_5)]: frequency of seeing/speaking to relatives/friends/neighbours.  Additional questions on help/support (practical, financial, emotional) from the SHARP^[[1]](#footnote-2)^ study[[97](#_ENREF_6)]. |
|  | *Neighbourhood characteristics* | Perceived neighbourhood safety from the British Household Panel Survey  Satisfaction with neighbourhood environment (general, environment, buildings, noise, parks, children’s play areas - Scottish Household Condition Survey. |
|  | *General health* | Health related quality of life – EQ-5D[[61-63](#_ENREF_7)]; chronic disease diagnoses; GP consultations (general, mental health) |
|  | *Alcohol and tobacco use* | Questions adapted from the Health Survey for England 2008[[94](#_ENREF_2)]7 |
|  | *Anthropometric* | Self-reported height and weight; waist circumference measured with tape measure during interview (self-report if refuse measurement) |
|  | *Sociodemographics* | Age; gender; ethnicity; nationality; marital status; housing tenure and duration of residency; educational attainment; personal and household income; employment status and occupation; household size and relationships; |
| Adolescent self-complete survey | *Sociodemographics* | Ethnicity; religion; duration of UK residency; household size and relationships; parental occupation; Family Affluence Scale[[73](#_ENREF_10)]; |
|  | *Healthy physical activity* | Physical Activity Questionnaire for Adolescents (PAQ-A)[[63](#_ENREF_11)] |
|  | *Healthy eating* | Short food frequency questionnaire (designed for this study) capturing weekly frequency of consumption of: breakfast; fruit; glass/small bottle of water; meal with parents/guardians; vegetables/salad; chips; sweets/chocolate; sugar sweetened drinks; high fat meal.  Questions to capture weekly frequency of: discussing food with parents and friends; worrying about body weight; going to bed hungry; planning meals. |
|  | *Mental wellbeing* | Strengths and Difficulties Questionnaire (SDQ)[[64](#_ENREF_12)]  Positive and Negative Affect Scale (PANAS)[[65](#_ENREF_13)]  Multidimensional Scale of Perceived Social Support (MSPSS)[[98](#_ENREF_14)]  Rosenberg Self-Esteem scale[[99](#_ENREF_15)]  Satisfaction with Life scale[[100](#_ENREF_16)] |
|  | *General Health* | Health service use (GP and dentist visits in previous year); hours of sleep; self-rated general health. |
|  | *Anthropometry* | Self-reported weight and height |
|  | *Smoking, alcohol, drug use* | Drug, tobacco and alcohol use questions taken from the RELACHS^[[2]](#footnote-3)^ study[[101](#_ENREF_17)] |
|  | *School environment* | Experience of violence at school and home – questions taken from SHEU^[[3]](#footnote-4)^ |
|  | *Home neighbourhood environment* | Perceived quality of neighbourhood services/facilities, safety, social cohesion – questions taken from RELACHS study[[101](#_ENREF_17)] |
|  | *Participation in Well London* | Awareness of and participation in Well London – respondent and family members |
|  | *Educational aspirations* | Expectations about GCSE, A-level results and going to university, peer and family support for education – taken from RELACHS study[[101](#_ENREF_17)] |
| Neighbourhood environmental audit | *Green spaces* | Number of communal green spaces, large parks and playgrounds |
|  | *Public amenities and services* | Number of off-street parking facilities, fountains, public toilets  Presence of Post Office, library, bank, gym, school, swimming pool, health centre (GP/dentist), church, community centre, pharmacy, betting shop, pawn brokers, pubs/bars. |
|  | *Cyclability* | Presence of cycle lanes |
|  | *Walkability* | Speed limit, number traffic calming measures, number of road crossing aids and overpasses/underpasses  Condition and width of pavements; size of roads (number of lanes of traffic) |
|  | *Food retail environment* | Types of food shops (groceries, prepared food, restaurants) including availability of fast food and vending machines holding sugar sweetened drinks and snacks |
|  | *Media environment* | Presence and number of advertisements for alcoholic drinks, sugar sweetened drinks, snack foods and for promotion of physical activity, healthy foods/diet, smoking cessation products and services |
|  | *Signs of social disorder and incivilities* | Presence and amount of: litter and broken glass; graffiti; vandalised facilities; broken windows; security measures; unattended dogs; large items dumped in public areas; dog foul; needles/syringes/condoms; empty alcohol bottles/cans; signs of home personalisation; greenery; neighbourhood watch signs |

1. Scotland's Housing And Regeneration Project (2002-2008) [↑](#footnote-ref-2)
2. Research with East London Adolescents: Community Health Survey [↑](#footnote-ref-3)
3. The Schools and Students Health Education Unit, [↑](#footnote-ref-4)
