# Supplementary material for: The Well London program - a cluster randomized trial of community engagement for improving health behaviors and mental wellbeing: baseline survey results
Source: Trials. 2012 Jul 6;13:105. doi: 10.1186/1745-6215-13-105 (PMC3441284; doi:10.1186/1745-6215-13-105)
Supplement: Additional file 2 — Statistical Analysis Plan for the Well London Cluster Randomised Trial [[7-30]]. [file 1745-6215-13-105-S2.docx]

Statistical Analysis Plan for the Well London Cluster Randomised Trial

[1. Trial outcomes 2](#_Toc325467689)

[**1.1.** **Hypotheses** 2](#_Toc325467690)

[**1.2.** **Description of quantitative data collection** 3](#_Toc325467691)

[**1.3.** **Primary outcomes** 9](#_Toc325467692)

[**1.4.** **Secondary outcomes** 10](#_Toc325467693)

[**1.5.** **Process measures (exposure variables)** 12](#_Toc325467694)

[**1.6.** **Potential confounding factors and effect modifiers** 13](#_Toc325467695)

[2. Plan of analysis for quantitative outcome data 25](#_Toc325467696)

[**2.1.** **Objectives** 25](#_Toc325467697)

[**2.2.** **Data structure** 25](#_Toc325467698)

[**2.3.** **Quality assurance** 25](#_Toc325467699)

[**2.4.** **Statistical methods** 26](#_Toc325467700)

[**2.4.1.** **Primary trial analysis** 26](#_Toc325467701)

[**2.4.2.** **Parameters** 33](#_Toc325467702)

[**2.4.3.** **Unadjusted analysis** 33](#_Toc325467703)

[**2.4.4.** **Adjusted analysis (covariates and baseline values)** 34](#_Toc325467704)

[**2.4.5.** **Exploratory analyses** 34](#_Toc325467705)

[**2.4.6.** **Multiple Imputation** 41](#_Toc325467706)

# Trial outcomes

- 1. **Hypotheses**

The hypotheses relate to differences between the intervention and control group at follow-up after three years of intervention delivery.

Primary Hypothesis

1. The Well London programme causes improvements in the healthy eating, healthy physical activity and mental wellbeing of adults and adolescents living in the target LSOAs.

Secondary Hypotheses – outcomes

1. Well London causes increases in social capital and community cohesion and improvements in residents’ perceptions of neighbourhood safety and quality.
2. Well London will cause reductions in crime related to anti-social behaviour and in incivilities (social disorder) in the target LSOAs.

Secondary Hypotheses – mechanisms, mediators and moderators

*Participation and exposure*

1. LSOAs with greater exposure to Well London projects will show greater area-level improvements in the healthy eating, healthy physical activity and mental wellbeing of adult and adolescent residents, where exposure is conceptualised in three ways: the amount of activities on offer; the prevalence of adult/adolescent participation in the LSOA; and the time at which the Well London Delivery Team volunteers were in place after the start of the programme.
2. Individuals with greater participation in Well London activities will be more likely to show individual-level improvements in healthy eating, physical activity and mental wellbeing.

*Mediators- individual*

1. The effects of the Well London intervention on healthy eating and physical activity levels are mediated by changes in mental wellbeing.

*Mediators – area level*

1. The effects of the Well London programme on the primary outcomes are mediated by the level of community cohesion in the LSOA, and by residents’ perceptions of neighbourhood quality and safety.
2. The effects of the Well London programme on the primary outcomes are mediated by the presence and intensity of signs of incivilities and social disorder at follow-up.

*Moderators- area level*

1. Areas with lower resident turnover will show greater improvements in the primary outcomes.
2. There will be greater improvements in physical activity in areas that: (i) are located closer to parks or large green spaces; (ii) are located closer to sports and leisure facilities; (iii) have a greater total area of green space within the LSOA; (iv) have better facilities for cycling (cycle lanes and storage); (v) have better provision for pedestrians (e.g. quality of pavements, road crossing aids, traffic calming measures).
3. There will greater improvements in healthy eating in areas that: (i) have a greater number of shops selling fruit and vegetables at baseline; (ii) are located closer to a large supermarket of food store; (iii) have fewer fast food restaurants at baseline.
   1. **Description of quantitative data collection**

The quantitative follow-up data for the trial analysis are being collected via:

Adults – household, interviewer-administered survey

Adolescents – self-complete survey, administered in school setting

Neighbourhood environmental data – field worker-completed structured audit tool

Crime reports – data request to London Metropolitan Police.

Table 1 shows the domains covered in each of the primary data collection tools.

One hundred adults were interviewed in each intervention and control LSOA; the household addresses were selected at random from the Post Office Address File and fieldworkers interview all eligible, consenting adults (aged 16 years or older) in each household. The total target sample size for the adolescent survey is 1200 respondents, with a target of 30 respondents per LSOA.

Table 1: Domains of data collected in the quantitative follow-up measurement tools

| **Data collection tool** | **Domains collected** | **Questions** |
| --- | --- | --- |
| Adult household survey | *Healthy physical activity* | International Physical Activity Questionnaire[[7](#_ENREF_7)] |
|  | *Healthy eating* | Food frequency questionnaire for fruit and vegetables adapted from Health Survey for England 2008[[8](#_ENREF_8)] |
|  | *Mental wellbeing* | General Health Questionnaire 12 item (GHQ12)[[9](#_ENREF_9)]; Warwick-Edinburgh Mental Wellbeing Scale[[10](#_ENREF_10)]; the Hope Scale[[5](#_ENREF_5)] |
|  | *Social capital* | Questions from the Office for National Statistics Social Capital Harmonised Question Set[[11](#_ENREF_11), [12](#_ENREF_12)]: frequency of seeing/speaking to relatives/friends/neighbours.  Additional questions on help/support (practical, financial, emotional) from the SHARP^^[[1]](#footnote-1)^^ study[[13](#_ENREF_13)]. |
|  | *Social cohesion* | Questions from the Office for National Statistics Social Capital Harmonised Question Set[[11](#_ENREF_11), [12](#_ENREF_12)]: social behaviour in public spaces (litter, drunkenness, vandalism, drug use); racial prejudice/violence; social/ethnic mixing; reciprocity amongst neighbours; trust. |
|  | *Collective efficacy* | Influencing decisions affecting local area. |
|  | *Neighbourhood characteristics* | Perceived neighbourhood safety from the British Household Panel Survey  Satisfaction with neighbourhood environment (general, environment, buildings, noise, parks, children’s play areas - Scottish Household Condition Survey. |
|  | *General health* | Health related quality of life – EQ-5D[[14](#_ENREF_14) ]; chronic disease diagnoses; GP consultations (general, mental health) |
|  | *Alcohol and tobacco use* | Questions adapted from the Health Survey for England 2008[[8](#_ENREF_8)] |
|  | *Anthropometric* | Self-reported height and weight; waist circumference measured with tape measure during interview (self-report if refuse measurement) |
|  | *Sociodemographics* | Age; gender; ethnicity; nationality; marital status; housing tenure and duration of residency; educational attainment; personal and household income; employment status and occupation; household size and relationships; |
|  | *Participation* | Participation in Well London activities – respondent and family members (detailed questions in intervention areas only; yes/no participation in Well London for control areas)  Participation in other activities related to health and mental wellbeing (both intervention and control areas) |
| Adolescent self-complete survey | *Sociodemographics* | School year; gender; Ethnicity; religion; duration of UK residency; household size and relationships; parental occupation; Family Affluence Scale[[15](#_ENREF_15)]; |
|  | *Healthy physical activity* | Physical Activity Questionnaire for Adolescents (PAQ-A)[[16](#_ENREF_16)] |
|  | *Healthy eating* | Short food frequency questionnaire (designed for this study) capturing weekly frequency of consumption of: breakfast; fruit; glass/small bottle of water; meal with parents/guardians; vegetables/salad; chips; sweets/chocolate; sugar sweetened drinks; high fat meal.  Self-report estimated portions of fruit and vegetables per day.  Questions to capture weekly frequency of: discussing food with parents and friends; worrying about body weight; going to bed hungry; planning meals. |
|  | *Mental wellbeing* | Strengths and Difficulties Questionnaire (SDQ)[[17](#_ENREF_17)]  Positive and Negative Affect Scale (PANAS)[[18](#_ENREF_18)]  Multidimensional Scale of Perceived Social Support (MSPSS)[[19](#_ENREF_19)]  Rosenberg Self-Esteem scale[[20](#_ENREF_20)]  Satisfaction with Life scale[[21](#_ENREF_21)] |
|  | *General Health* | Health service use (GP and dentist visits in previous year); hours of sleep; self-rated general health. |
|  | *Anthropometry* | Self-reported weight and height |
|  | *Smoking, alcohol, drug use* | Drug, tobacco and alcohol use questions taken from the RELACHS^^[[2]](#footnote-2)^^ study[[22](#_ENREF_22)] |
|  | *School environment* | Experience of violence at school and home – questions taken from SHEU^^[[3]](#footnote-3)^^ |
|  | *Home neighbourhood environment* | Perceived quality of neighbourhood services/facilities, safety, social cohesion – questions taken from RELACHS study[[22](#_ENREF_22)] |
|  | *Participation in Well London* | Awareness of and participation in Well London – respondent and family members |
|  | *Educational aspirations* | Expectations about GCSE, A-level results and going to university, peer and family support for education – taken from RELACHS study[[22](#_ENREF_22)] |
| Neighbourhood environmental audit | *Green spaces* | Number of communal green spaces, large parks and playgrounds |
|  | *Public amenities and services* | Number of off-street parking facilities, fountains, public toilets  Presence of Post Office, library, bank, gym, school, swimming pool, health centre (GP/dentist), church, community centre, pharmacy, betting shop, pawn brokers, pubs/bars. |
|  | *Cyclability* | Presence of cycle lanes and cycle storage facilities. |
|  | *Walkability* | Speed limit, number traffic calming measures, number of road crossing aids and overpasses/underpasses  Condition and width of pavements  Size of roads (number of lanes of traffic) |
|  | *Food retail environment* | Types of food shops (groceries, prepared food, restaurants) including availability of fast food and vending machines holding sugar sweetened drinks and snacks  Number of shops selling fruit and/or vegetables and number of fruit and vegetable items available in each shop |
|  | *Media environment* | Presence and number of advertisements for alcoholic drinks, sugar sweetened drinks, snack foods and for promotion of physical activity, healthy foods/diet, smoking cessation products and services |
|  | *Signs of social disorder and incivilities* | Presence and amount of: litter and broken glass; graffiti; vandalised facilities; broken windows; security measures; unattended dogs; large items dumped in public areas; dog foul; needles/syringes/condoms; empty alcohol bottles/cans; signs of home personalisation; greenery; neighbourhood watch signs |

- 1. **Primary outcomes**

The Well London programme was designed to improve healthy eating, healthy physical activity and mental health and wellbeing in adults and young people (adolescents). Measures for each of these health behaviours and outcomes will be assessed as primary outcomes in the trial. The primary outcomes are:

*Healthy eating*

Adults – binary classification according to whether the respondent consumes at least five portions of fruit or vegetables per day (as per recommendations from the Chief Medical Officer); the consumption of fruit and vegetables are measured using a food frequency questionnaire, adapted from the health survey for England, administered within the household survey;

Adolescents – binary classification according to whether the respondent consumes at least five portions of fruit or vegetables per day; measured as self-reported estimated portions of fruit and vegetables per day in a single questionnaire item.

*Physical Activity*

Adults – binary classification according to whether the respondent completes at least five sessions of moderate intensity physical activity per week each lasting at least 30 minutes (as per Chief Medical Officer guidelines[[23](#_ENREF_23)])^^[[4]](#footnote-4)^^; levels of physical activity are measured using the International Physical Activity Questionnaire (IPAQ)[[7](#_ENREF_7)] administered in the household survey.

Adolescents – score from the Physical Activity Questionnaire for Adolescents (PAQ-A) [[24](#_ENREF_24)] (ranging from 1-inactive to 5-very active) administered in the adolescent self-complete survey.

*Mental health and wellbeing*

Adults (mental ill health) – normal mental health score (binary classification according to the validated cut-off) on the 12-item General Health Questionnaire (GHQ12).

Adults (positive mental wellbeing) – continuous score on the Warwick Edinburgh Mental Wellbeing Scale (WEMWBS)[[10](#_ENREF_10)] administered in the household survey; the WEMWBS is designed as a measure of population-level positive mental wellbeing and does not have a recommended cut-off for “normal wellbeing”; authors suggest categorising respondents in terms of the number of standard deviations between their individual score and the overall mean to give three categories (poor, average, good wellbeing).

Adolescents (mental ill health) – normal score (binary classification) on the adolescent Strengths and Difficulties Questionnaire (SDQ)[[17](#_ENREF_17)] administered in the adolescent self-complete survey.

Adolescents (general mental wellbeing) – continuous score for positive affect sub-scale (positive mood) on the Positive and Negative Affect Scale (PANAS)[[18](#_ENREF_18)]; continuous score for negative affect sub-scale (negative mood) on the PANAS; the PANAS is administered in the adolescent self-complete survey.

All primary outcomes will be measured at follow-up and compared between intervention and control areas (see section 5.3.3).

The primary trial outcomes are summarised in Table 2.

- 1. **Secondary outcomes**

*Social and community processes*

Process evaluation from CADBE and Wellnet events suggests that there may have been changes in the community and social processes in the Well London intervention areas, particularly relating to community cohesion (knowing and speaking to neighbours more)and anti-social behaviour and related criminal offences. These phenomena are being systematically captured in the adult household survey and in the neighbourhood environmental audit and are therefore amenable to further investigation. The following social and community processes will be examined:

- - - Adult social capital – measured as contact with friends and neighbours (4 questionnaire items – adult household survey)
    - Adult social support – measured as the number of people who would provide help (3 questionnaire items – adult household survey)
    - Social integration – measured as residents from different backgrounds “getting on” and residents trusting each other (2 questionnaire items – adult household survey)
    - Collective efficacy – measured as residents improving the neighbourhood together and helping each other out; individual activism (3 questionnaire items – adult household survey)
    - Volunteering activities (1 questionnaire item – adult household survey)
    - Anti-social behaviour reported by residents – measured as rating the level of problem with particular behaviours (6 questionnaire items – adult household survey)
    - Signs of anti-social behaviour measured by fieldworkers – measured as frequency of observing specific items (10 items (combined as a score) – environmental audit tool)
    - Fear of crime – measured as perceived neighbourhood safety (2 questionnaire items – adult household survey)
    - Reported crime related to anti-social behaviours – crime report data from the London Metropolitan Police.

The questionnaire items used to measure the social and community processes are summarised in Table 4.

*Health behaviours and mental wellbeing*

Several of the binary primary outcomes are based on a continuous score. The continuous scores may be more sensitive to change than the binary classifications and allow examination of changes in the distribution of these outcomes. The difference in population mean score and distribution between intervention and control groups will therefore also be examined for:

- - - Adolescent negative mental health - SDQ
    - Adult negative mental health - GHQ12
    - Adult physical activity - IPAQ
    - Adult healthy eating - Portions of fruit and vegetables per day

In the 2004 guidelines on physical activity, the Chief Medical Officer for England has made two recommendations on levels of healthy physical activity: five sessions of moderate intensity physical activity per week lasting at least 30 minutes are necessary for general health; 60 minutes of moderate intensity physical activity per day is required for obesity prevention[[23](#_ENREF_23)]. Binary classification of adults meeting this second requirement for obesity prevention will be assessed as a secondary outcome at follow-up. In July 2011 the Chief Medical Officers for England, Wales, Scotland and Northern Ireland issued joint guidance on physical activity for health, which retained the recommendation of at least 30 minutes of vigorous physical activity on five or more days per week (accumulated in sessions of at least 10 minutes at a time)[[25](#_ENREF_25)]. However, expert review of the current evidence base suggested that there may be equivalent benefits from simply accumulating a total of at least 150 minutes of moderate intensity physical activity per week without the requirement that it be spread over five days, meaning that three sessions of 50 minutes per week would now meet the guidelines. Whilst these new guidelines were introduced after the end of the Well London programme delivery, we will include meeting the recommended 150 minutes of moderate intensity physical activity per week (or metabolic equivalent) as a binary secondary outcome.

The Eatwell project in Well London focussed on increasing consumption of fruit and vegetables and also on decreasing intake of unhealthy foods that are high in fat, sugar or salt. Consumption of unhealthy foods in adults will be assessed as a secondary outcome to complement the primary outcome relating to consumption of fruit and vegetables. The food groups that will be assessed are: fried foods; savoury snacks (crisps, salted nuts); cakes and puddings; sweets and chocolates; sugar sweetened soft drinks. In adolescents, consumption of chips, sweets or chocolate and sugar sweetened soft drinks will be assessed. For both the adults and adolescents an unhealthy eating score will be created from the Likert scale responses indicating frequency of consumption, as described in Table 3. The secondary trial outcomes are summarised in Table 3.

- 1. **Process measures (exposure variables)**

The activities delivered within the Well London projects were open to all LSOA residents but attendance was voluntary; some activities had no limit on numbers of participants (e.g. Community Feasts) whereas others had limited places (e.g. Cook and Eat sessions); some activities were delivered throughout the duration of the three year programme, but some were delivered only during discrete time periods (see Figure 1). Therefore, whilst all intervention LSOAs received the Well London programme, not all residents will have had contact with the programme.

Several process measures have been collected for Well London. Individual level exposure is collected as participation in Well London activities reported in the adult household survey and in the adolescent self-complete questionnaire. In the adult survey only, respondents are also asked if other members of their household participated in Well London activities.

The individual-level participation measures can be aggregated to provide a neighbourhood-level prevalence of participation. We hypothesise that because changes in community processes may have occurred, and may be a pathway through which Well London impacts on the primary outcomes, neighbourhood-levels of participation will be an important process measure of these community-level phenomena and represent a different construct to individual-level participation.

At the neighbourhood level there are three further process measures: (i) the number of quarters of project activity on offer (see Section 2.6 and Figure 1); and (ii) the programme quarter in which the Well London Delivery Team was recruited, trained and became active (see Figure 1 for variability in commencement of Well London Delivery Team activities across the intervention LSOAs.) The number of quarters of project activity crudely represents the opportunities for engagement with the Well London programme. There may be qualitative differences between the Phase 1 and Phase 2 boroughs because the experience of setting-up and delivering Well London in the Phase 1 boroughs informed actions in the Phase 2 boroughs. The Well London Delivery Teams were a key component of the programme; these volunteers were trained in community engagement and activation and were responsible for advertising and promoting Well London activities to residents and therefore are likely to have had a substantial impact on levels of participation.

The measure of exposure using the quarters of projects on offer is a crude proxy for the amount of delivery time: it does not take account of the likely mechanistic difference between a one-off event in comparison to a regular weekly activity. These measures also do not take account of qualitative differences noted in the success of delivery e.g. differences in baseline levels of motivation to engage with and take part in Well London activities between the areas or differing success of the Well London Delivery Teams.

- 1. **Potential confounding factors and effect modifiers**

Whilst the analysis of the baseline surveys indicates that the intervention and control LSOAs are broadly comparable there may be differences within each pair of LSOAs that could increase the between-cluster variation and reduce the precision of the effect estimates[[26](#_ENREF_26)]. Therefore age, gender, ethnicity, socioeconomic status (employment status for adults, family affluence scale for adolescents) and educational attainment (adults only) will be treated as potential confounders.

The potential for effect modification of the main trial outcomes will be examined using subgroup analyses across age group, gender, ethnicity, socioeconomic status, educational attainment (adults only). Additionally, neighbourhood-level analyses will be used to examine effect modification by neighbourhood characteristics (e.g. food environment, amount of green space) and by population turnover in the LSOA (LSOA-level mean duration of residency).

Table 2**:** Primary outcome measures

| Outcome type | Age group | Outcome | Indicator | Measurement tool | Data collection tool |
| --- | --- | --- | --- | --- | --- |
| Primary | Adults | Healthy eating | Binary – consumption of 5 or more portions of fruit and vegetables per day | Food frequency questionnaire | Adult household survey |
| Primary | Adults | Healthy physical activity | Binary – doing five or more sessions of moderate intensity physical activity per week lasting at least 30 mins | International Physical Activity Questionnaire | Adult household survey |
| Primary | Adults | Mental health – negative | Binary – score above threshold for normal mental health | 12 item General Health Questionnaire | Adult household survey |
| Primary | Adults | Mental health – positive wellbeing | Continuous - score | Warwick-Edinburgh Mental Wellbeing Scale | Adult household survey |
| Primary | Adolescent | Healthy eating | Binary – consumption of 5 or more portions of fruit and vegetables per day | Single questionnaire item requesting self-estimated portions per day | Adolescent self-complete survey |
| Primary | Adolescents | Healthy physical activity | Continuous - IPAQ score | Physical Activity Questionnaire for Adolescents | Adolescent self-complete survey |
| Primary | Adolescents | Mental health - negative | Binary – score above threshold for normal mental health | Strengths and Difficulties Questionnaire | Adolescent self-complete survey |
| Primary | Adolescents | Mental health – positive wellbeing | Continuous – positive affect score and negative affect score | Positive and negative affect scale | Adolescent self-complete survey |

Table 3**:** Secondary outcome measures

| Outcome type | Age group | Outcome | Indicator | Measurement tool | Data collection |
| --- | --- | --- | --- | --- | --- |
| Secondary | Adults | Unhealthy eating | Continuous – score comprised of mean likert scale points for frequency of consumption of: fried foods; savoury snacks (crisps, salted nuts); cakes and puddings; sweets and chocolates; sugar sweetened soft drinks | Food frequency questionnaire  Scale points:  *6 or more times per week (5)*  *3-5 times per week(4)*  *1-2 times per week(3)*  *Less than once a week(2)*  *Rarely or never(1)* | Adult household survey |
| Secondary | Adults | Healthy eating | Continuous – number of portions of fruit and vegetables per day | Food frequency questionnaire | Adult household survey |
| Secondary | Adolescents | Unhealthy eating | Continuous – score comprised of mean likert scale points for frequency of consumption of sweets and chocolate; chips; and sugar sweetened drinks | Scale points:  *Every day without exception (5)*  *Almost every day(4)*  *3-4 times a week(3)*  *1-2 times a week(2)*  *Hardly ever(1)* | Adolescent self-complete survey |
| Secondary | Adults | Healthy physical activity | Binary – doing 60 minutes of physical activity per day | International Physical Activity Questionnaire | Adult household survey |
| Secondary | Adults | Healthy physical activity | Binary – doing 150 minutes of moderate intensity physical activity per week | International Physical Activity Questionnaire | Adult household survey |
| Secondary | Adults | Healthy physical activity | Continuous – MET-minutes^^[[5]](#footnote-5)^^ of activity per week | International Physical Activity Questionnaire | Adult household survey |
| Secondary | Adults | Mental health – negative | Continuous – GHQ12 score | 12 item General Health Questionnaire | Adult household survey |
| Secondary | Adolescents | Mental health - negative | Continuous – SDQ score | Strengths and Difficulties Questionnaire | Adolescent self-complete survey |
| Secondary | Adults | Social capital | Contact with friends and neighbours | ONS social capital harmonised question set | Adult household survey |
| Secondary | Adults | Social support | Help available for practical, financial and emotional problems | ONS social capital harmonised question set | Adult household survey |
| Secondary | Adults | Social integration | Residents’ perceptions that neighbours of different backgrounds get along and that neighbours can be trusted | ONS social capital harmonised question set | Adult household survey |
| Secondary | Adults | Collective efficacy | Residents’ perceptions that neighbours help each other and work together to improve the neighbourhood;  Binary - involvement in activism on local issues | ONS social capital harmonised question set | Adult household survey |
| Secondary | Adults | Volunteering | Binary - participation in volunteering activities | ONS social capital harmonised question set | Adult household survey |
| Secondary | Adults | Antisocial behaviour | Reported by residents | ONS social capital harmonised question set | Adult household survey |
| Secondary | Adults | Antisocial behaviour | Coverage across the LSOA of signs of antisocial behaviour recorded by fieldworkers completing the environmental audit |  | Neighbourhood environmental audit |
| Secondary | Adults | Fear of crime | Residents’ perceptions of neighbourhood safety |  | Adult household survey |
| Secondary | Adults | Occurrence of crimes | Continuous – annual rate per capita of Police-reported crimes (criminal damage; violence against the person; drugs; robbery & burglary) |  | London Metropolitan Police |

Table 4**:** Questionnaire items used to measure social and community processes and outcome measures for the analysis

| Social / Community Process | Indicator | Questionnaire items | Response structure | Data collection | Outcome measure |
| --- | --- | --- | --- | --- | --- |
|  |  |  |  |  |  |
| Social networks | Contact with friends and neighbours | How often do you:   - - 1. Meet up with friends     2. Speak to friends on the phone     3. Write to friends     4. Speak to neighbours | Most days; once a week or more; once or twice a month; less often than once a month; never; don’t know | Adult household survey | Score the responses to indicate approximate number of days per month  Most days=28  Once a week or more=12  Once or twice a month=2  Less often than once a month=0.5  Never=0  Don’t know = treat as missing  Sum the scores across the domains to give a total relative frequency of social contact events |
| Social support | Help provided | How many people outside your home could you ask for the following kinds of help:   1. Buy groceries if you are unwell 2. Lend you money for a few days 3. Give advice and support in a crisis | None; one or two; more than two; would not ask; | Adult household survey | Score the responses:  None=0  One or two = 1  More than two=2  Would not ask = 0  Don’t know=missing  Prefers not to say = missing    Sum scores across questions to give a social support score with range 0-6 |
| Social integration | Residents’ perceptions of neighbour interaction | Would you say that:   - 1. Most of the people in your neighbourhood can be trusted   2. Some can be trusted   3. A few can be trusted   4. No-one can be trusted   5. Just moved here   6. Don’t know   7. Prefers not to say |  | Adult household survey | Separate binary outcomes:  Trust = most or some can be trusted vs. other responses |
|  |  | To what extent do you agree or disagree that this neighbourhood is a place where people from different backgrounds get on well together? | Definitely agree; tend to agree; tend to disagree; definitely disagree; don’t know; too few people in the neighbourhood; all same background |  | Different backgrounds get on = definitely or tend to agree vs. other responses |
|  |  | How much of a problem is people being attacked or harassed because of their skin colour, ethnic origin or religion? | Very big problem; fairly big problem; not a very big problem; it happens but it’s not a problem; not a problem at all; don’t know |  | Racial harassment = very or fairly big problem vs. other responses |
| Collective efficacy | Residents’ perceptions of neighbours mutual help and working together; involvement in activism on local issues | To what extent do you agree or disagree that people in this neighbourhood pull together to improve the neighbourhood? | Definitely agree; tend to agree; tend to disagree; definitely disagree; don’t know; nothing needs improving | Adult household survey | Separate binary outcomes:  People pull together = definitely or tend to agree vs. other responses |
|  |  | Is this a neighbourhood in which people do things together and try to help each other, or one in which people mostly go their own way? | Help each other; go own way; mixture; don’t know |  | Help each other = health each other vs. other responses |
|  |  | In the last 12 months have you taken any of the following actions in an attempt to solve a problem affecting people in your local area? | Contacted a local radio/television station or newspaper; contacted the appropriate organisation such as the council; contacted a local councillor or MP; attended a public meeting or neighbourhood forum to discuss local issues; attended a tenants’ or local residents’ group; attended a protest meeting or joined an action group; helped organise a petition on a local issue; no local problems; none of these; don’t know; none of the above |  | Binary: Taken any action vs. no action |
| Volunteering | Involvement in volunteering activities | During the last 12 months have you given any unpaid help to any groups, clubs or organisations in any of these ways? | Raising or handling money/taking part in a sponsored event; leading the group/member of a committee; organising or helping run an activity or event; visiting people; befriending or mentoring people; giving advice/information/counselling; secretarial/admin/clerical work; providing transport/driving; representing; campaigning; other practical help; any other help; none of the above | Adult household survey | Binary: Involvement in any activity vs. no involvement |
| Antisocial behaviour | Residents’ perceptions of antisocial behaviour | I am going to read out a list of problems which some people face in their neighbourhood. For each one, please can you tell me how much of a problem it is:  How much of a problem are people being drunk or rowdy in public places?  How much of a problem is rubbish or litter lying around?  How much of a problem are vandalism, graffiti and other deliberate damage to  property or vehicles?  How much of a problem are people using or dealing drugs?  How much of a problem are teenagers hanging around on the street?  How much of a problem are troublesome neighbours? | Very big problem; fairly big problem; not a very big problem; it happens but it’s not a problem; not a problem at all; don’t know | Adult household survey | Binary indicator for each question:  **Very or fairly big problem vs. other responses**  Sum binary scores across the questions to give a perceived antisocial behaviour score ranging between 0 and 6 |
| Antisocial behaviour | Signs of antisocial behaviour observed by field workers | When you walked around this segment did you see:  Litter of broken glass  Graffiti  Broken or vandalised facilities  Broken windows  Unattended dogs  Large items dumped in public areas (furniture/cars)  Dog foul  Needles, syringes or condoms  Empty beer cans or alcohol bottles  Sex paraphernalia (condoms, cards) | None; little; moderate amount; a lot | Neighbourhood environmental audit | Score none=0, little=1, moderate=2, a lot=3  Calculate the mean score for each domain (i.e. litter, graffiti etc.) across the surveyed segments in the LSOA. Sum the domain mean scores for the LSOA and standardise to range between 0 and 100 |
| Fear of crime | Residents’ perceptions of neighbourhood safety | How safe do you feel generally when you are walking outside alone in this neighbourhood during the daytime?  How safe do you feel when you are walking outside in this neighbourhood alone after dark? | Very safe; fairly safe; a bit unsafe; very unsafe; never out alone | Adult household survey | Separate binary outcomes:  **Very or fairly safe vs. other responses** |
| Occurrence of crime | Crimes reported to police | Criminal Damage  Violence against the person  Drugs  Robbery & Burglary |  | London Metropolitan Police | Overall crime rate |

#

1. **Plan of analysis for quantitative outcome data**
   1. **Objectives**

The primary objective of the Well London trial analysis is to measure the effect of the Well London community development intervention on levels of healthy eating, physical activity and mental wellbeing in adults and adolescents.

Secondary objectives are to:

- - - Examine evidence for differential effects of Well London in population subgroups;
    - Measure the effects of the Well London intervention on broader social outcomes related to community cohesion, social capital, crime and neighbourhood safety;
    - Examine the moderating effects of levels of intervention exposure, resident turnover and neighbourhood characteristics (e.g. food environment, incivilities, amount of green space) on intervention effects;
    - Examine the mediating effects of social capital, perceived neighbourhood safety and quality, and community cohesion on area-level intervention effects;
    - Examine the mediating effects of mental wellbeing on individual-level intervention effects.
  1. **Data structure**

The experimental unit is the LSOA, with intervention and control LSOAs pair-matched within London boroughs.

The unit of observation for the trial is the individual resident living within the LSOAs. Respondents for the adult survey were sampled by household address, with every eligible, consenting adult (aged 16 years or older) interviewed in each responding household. The adult survey data therefore have four levels: London Borough (matching); LSOA; household; individual respondents. The adolescent survey data also has four levels: London Borough; LSOA; school, individual respondents. Only adolescents living within the target LSOAs were invited to complete the adolescent survey at their school. The number of schools surveyed per borough ranged from one to seven, with the median number being three (includes the intervention and control LSOA in each borough). The number of pupils surveyed within each school ranged from one to 78, with the median at 13 pupils.

Four datasets will be used for the quantitative outcome analysis: the adult household survey; the adolescent school-based survey; the neighbourhood environmental audit; crime reports from the London Metropolitan Police.

- 1. **Quality assurance**

All of the primary and secondary outcomes and exploratory analyses for the trial are specified in detail in this document. The analyses will be unblinded, with one primary statistician running all analyses and a supporting statistician independently checking and validating all of the primary outcome analyses. The analysis code for Stata will be developed before the follow-up survey database is locked and data extracted for analysis, as a further measure to prevent any researcher bias.

- 1. **Statistical methods**
     1. **Primary trial analysis**

The primary analysis for the adult outcomes will be a matched analysis, adjusted for key sociodemographic and socioeconomic confounders, using complete cases (defined as those individuals who completed all outcome score items in the questionnaire and provided the key confounder information). The methods for the adjusted analysis are presented in section 4.4.4. Table 5 shows the high levels of completion of key analysis variables in the follow-up survey (data extract taken 22.11.11). A large number of participants refused to provide details of household and personal income, therefore these variables will not be used in the analyses. The completion rate for the primary outcomes is estimated to be at least 95% at the end of the survey; the individual scale items have high completion at 99% or higher (see Table 6), but until the data are fully extracted from the survey database and the scores computed in the trial analysis, the exact level of missing data in the calculated scores cannot be determined.

The primary analysis for the adolescent survey will be a matched analysis, adjusted for key sociodemographic and socioeconomic confounders (as per section 4.4.4.), using multiple imputation to account for missing data. The levels of missing data in the individual questionnaire items are very similar in the preliminary data extract from the adolescent follow-up survey (first 400 completed questionnaires) and the full baseline survey (see Figures 2a to 2e below). The methods for the multiple imputation are described in section 4.4.6.

The primary analyses will be adjusted for key sociodemographic variables and baseline LSOA-level outcome prevalence / means (see section 4.4.4.) to reduce between-cluster variation and therefore increase the power to detect effects.

Table 5: Completeness of responses to key auxiliary analysis variables in the adult household survey (N=2956; data extract from survey database 22.11.11)

|  | Percent of respondents providing information | |
| --- | --- | --- |
| Variable | Baseline | Follow-up survey |
| Age | 99.9 | 100.0 |
| Gender | 98.3 | 100.0 |
| Ethnicity | 97.5 | 99.5 |
| Employment status | 93.3 | 97.8 |
| Educational attainment | 88.9 | 98.8 |
| Household income | 44.7 | 49.0 |
| Personal income | 49.3 | 61.4 |
| Ease of managing on household income | 93.2 | 93.3 |
| Housing tenure | 93.4 | 98.8 |

Table 6: Completeness of responses to the primary outcome scores for the adult household survey (N=2956; data extract from survey database 22.11.11)

|  | Percent of respondents providing information | |
| --- | --- | --- |
| Variable | Baseline | Follow-up survey |
| IPAQ |  |  |
| Overall score | 80.6 | - |
| Individual items |  |  |
| Days of vigorous activity in last 7 days | 91.1 | 100.0 |
| Moderate activity (day) | 90.4 | 99.9 |
| Walking (day) | 92.1 | 99.8 |
| Daily sitting - hours | 71.0 | 99.9 |
| Daily sitting - minutes | 70.4 | 99.6 |
| Five-a-week classification | 85.9 | - |
| Food Frequency Questionnaire (24 hour recall) |  |  |
| Overall portions of fruit or vegetables (for five-a-day classification) | 93.4 | - |
| Individual items |  |  |
| Salad | 98.9 | 99.9 |
| Pulses | 98.1 | 99.8 |
| Vegetables | 98.3 | 99.8 |
| Vegetable dishes | 97.0 | 99.0 |
| Fruit juice | 98.0 | 99.9 |
| Fresh fruit | 98.7 | 99.9 |
| Dried fruit | 98.9 | 99.9 |
| Frozen/tinned fruit | 98.7 | 99.9 |
| Fruit dishes | 97.5 | 99.4 |
| GHQ12 |  |  |
| Item 1 | 97.8 | 99.9 |
| Item 2 | 98.6 | 99.8 |
| Item 3 | 97.6 | 99.8 |
| Item 4 | 98.5 | 99.9 |
| Item 5 | 97.4 | 99.9 |
| Item 6 | 98.8 | 99.7 |
| Item 7 | 98.2 | 99.9 |
| Item 8 | 97.9 | 99.8 |
| Item 9 | 98.9 | 99.8 |
| Item 10 | 98.2 | 100.0 |
| Item 11 | 97.1 | 99.8 |
| Item 12 | 98.2 | 99.9 |
| Warwick Edinburgh Mental Wellbeing Scale |  |  |
| Item 1 | - | 99.6 |
| Item 2 | - | 99.7 |
| Item 3 | - | 99.8 |
| Item 4 | - | 99.7 |
| Item 5 | - | 99.7 |
| Item 6 | - | 99.5 |
| Item 7 | - | 99.3 |
| Item 8 | - | 99.2 |
| Item 9 | - | 99.6 |
| Item 10 | - | 99.9 |
| Item 11 | - | 100.0 |
| Item 12 | - | 99.8 |
| Item 13 | - | 99.9 |
| Item 14 | - | 99.8 |
| Item 15 | - | 99.9 |
| Item 16 | - | 99.9 |
| Item 17 | - | 100.0 |
| Item 18 | - | 99.9 |
| Item 19 | - | 100.0 |
| Item 20 | - | 99.0 |
| Item 21 | - | 100.0 |
| Item 22 | - | 99.9 |
| Item 23 | - | 99.8 |
|  | - |  |

Figure 1: Completeness of responses in the adolescent baseline and follow-up surveys – gender, ethnicity, family affluence scale.


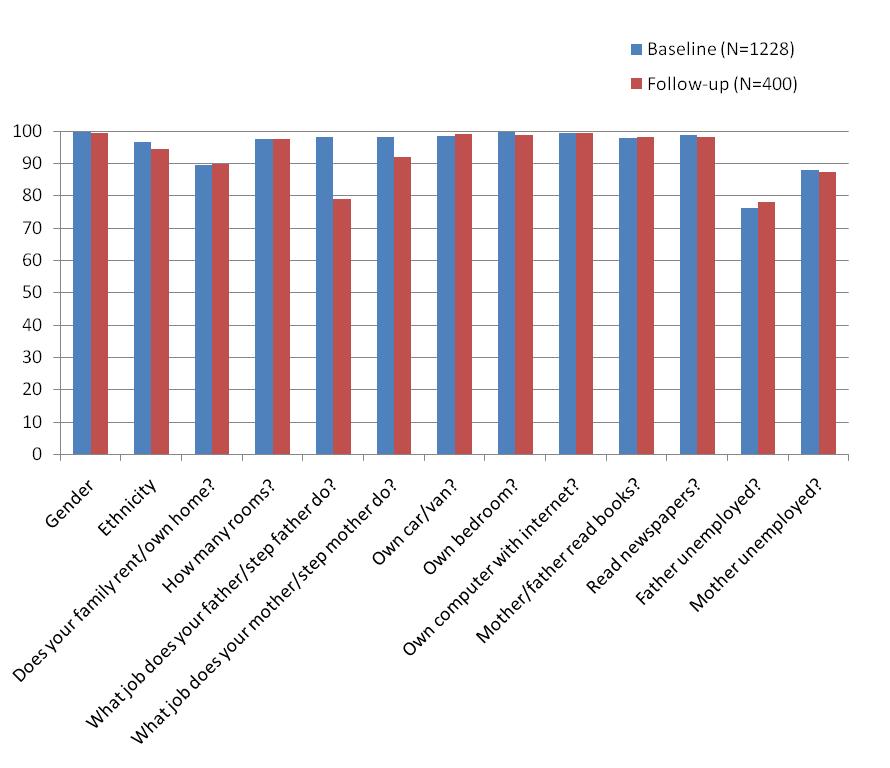


Figure 2: Completeness of responses in the adolescent baseline and follow-up surveys – PAQ-A


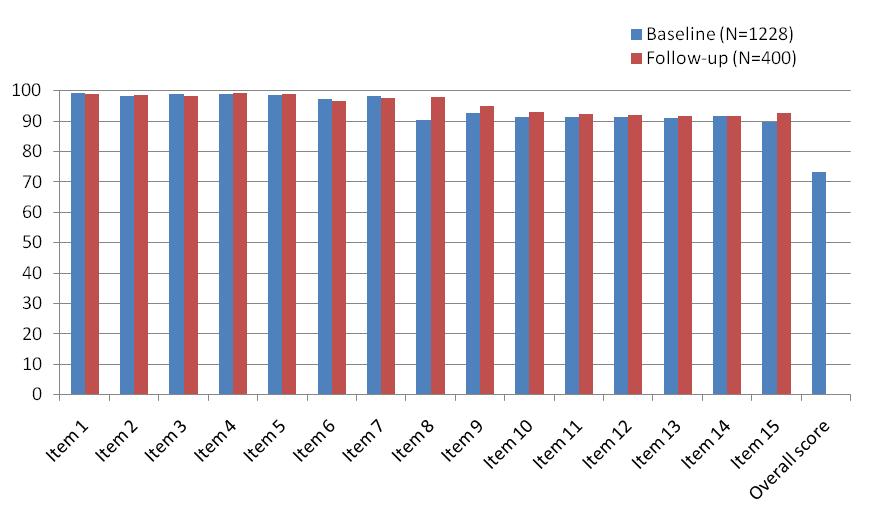


Figure 3: Completeness of responses in the adolescent baseline and follow-up surveys - SDQ


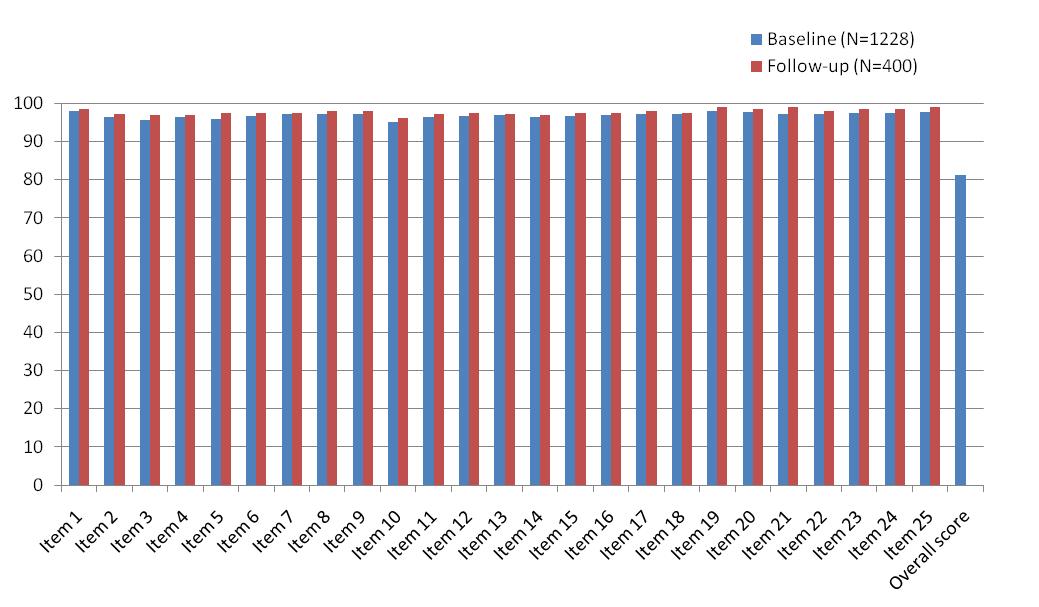


Figure 4: Completeness of responses in the adolescent baseline and follow-up surveys – PANAS


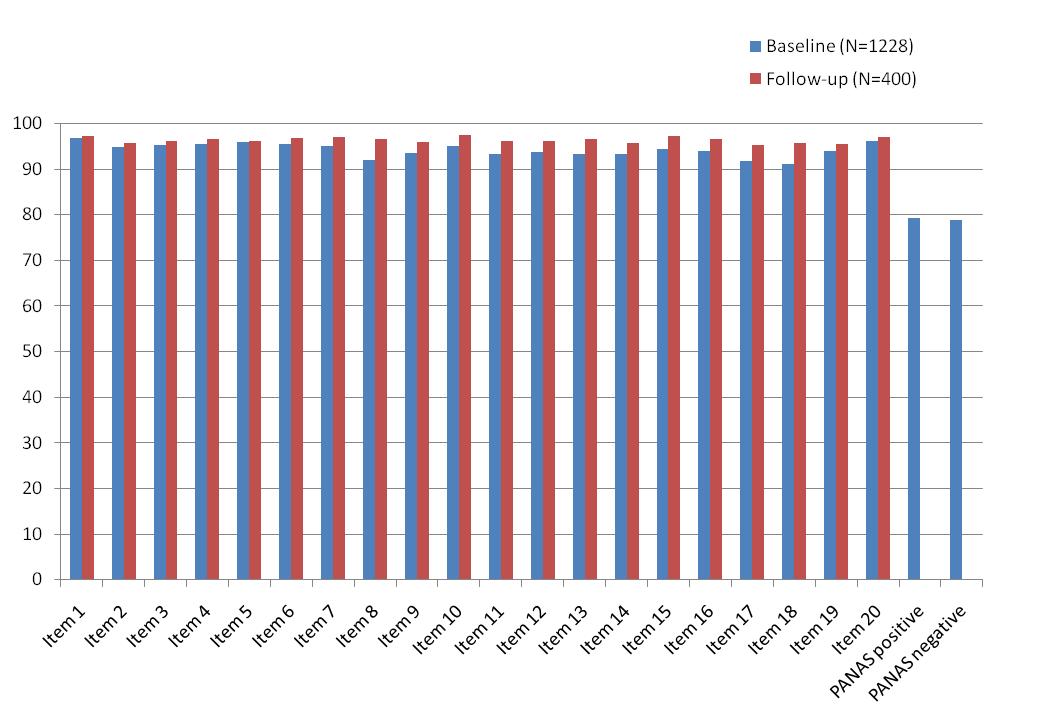


Figure 5: Completeness of responses in the adolescent baseline and follow-up surveys – Dietary items


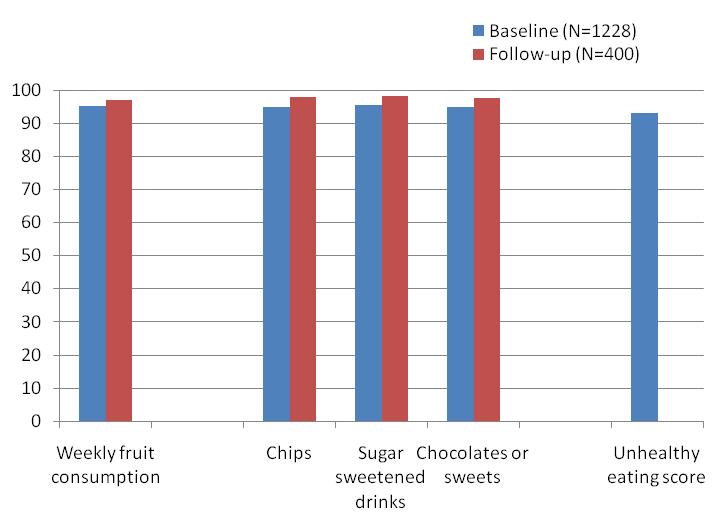


- - 1. **Parameters**

*Continuous outcomes*

Effects on continuous outcomes will be assessed using the mean difference between the intervention and control clusters. The parameter will be calculated as the unweighted average of the pair-specific difference in the mean outcome between the intervention and control LSOA in each borough:

$$\frac{1}{p}\sum_{j} {MD}_{j}$$

where *j* indicates the matched pair; *i*=0 in the control LSOA and *i*=1 in the intervention LSOA; *p* indicates the number of matched pairs; and:

$${MD}_{j}=m_{1j}-m_{0j}$$

where *m* is the LSOA-specific mean outcome.

*Binary outcomes*

Effects on binary outcomes will be assessed using the risk ratio between the intervention and control clusters. The parameter will be calculated as the exponent of the unweighted average of the pair-specific log risk ratios:

$$exp\left[ \frac{1}{p}\sum_{j} l_{1j}-l_{0j} \right]$$

where *j* indicates the matched pair; *i*=0 in the control LSOA and *i*=1 in the intervention LSOA; *p* indicates the number of matched pairs; and *l* is the log of the proportion of individuals with the outcome of interest.

- - 1. **Unadjusted analysis**

The average of the pair-specific (within borough) mean differences in continuous outcomes and risk ratios for binary outcomes will be calculated as described in section 4.4.1 using the multiply imputed datasets. Effect estimates will be calculated for all of the primary and secondary outcomes shown in Table 2 and Table 3.

The paired t-test with 19 degrees of freedom will be used to test the null hypothesis that the mean difference is zero for continuous outcomes and that the risk ratio is equal to one for binary outcomes. A 95% confidence will be calculated for each effect estimate using the formula:

$$\bar{h}\pm t_{v, 0.025}\times\frac{s_{m}}{\sqrt{c}}$$

Where $\bar{h}$ is the average mean difference or average difference in log risk across the pairs of intervention and control LSOAs;*t_v_*_,0.025_ is the upper 2.5% point of the t distribution; c is the number of matched pairs; *s_m­_* is the empirical standard deviation of the differences across the matched pairs and is calculated as:

$$s_{m}= \sqrt{\frac{1}{c-1} \sum_{j} {(h_{j}-\bar{h})}^{2}}$$

where *h_j_* is the difference in mean or log risk in the *j*^th^ matched pair.

- - 1. **Adjusted analysis (covariates and baseline values)**

Effect estimates for the primary outcomes and secondary outcomes will be adjusted for the distribution of the following individual-level characteristics:

- - - Age (adults); school year (adolescents)
    - Gender
    - Ethnicity
    - Highest achieved educational qualification (adults only
    - Employment status (adults); family affluence scale (adolescents).

Adjustment for covariates will be achieved in a two-stage process as described by Hayes and Moulton[[26](#_ENREF_26)]. A regression model (linear for continuous outcomes, logistic for binary outcomes) will be fitted to the individual-level outcomes, including the variables listed above and an indicator for the matched pairs of LSOAs, but no indicator for intervention/control status. The expected LSOA-specific means/risks will be extracted from the regression model and used to calculate an adjusted effect estimate which will then be used in the t-test as described in Section 4.4.3.

Where there are appropriate baseline values available, the LSOA-level mean or proportion of the outcome measured in the baseline cross-sectional survey will also be included as a covariate. The adult healthy eating and physical activity outcomes and the adolescent physical activity and mental wellbeing outcomes were measured in the baseline survey, therefore the LSOA-level prevalence or mean will be used for adjustment. The adult mental wellbeing outcomes (WEMWBS and GHQ12) and the adolescent healthy eating outcome were not measured at baseline, therefore other similar indicators will be used to adjust for baseline status (Hope scale score and prevalence of self-reported anxiety or depression (from the EQ5D) for adult mental wellbeing; weekly consumption of fruit for adolescent healthy eating.) One degree of freedom will be removed from the LSOA-level t-test for each LSOA-level continuous variable or binary outcome variable used for baseline adjustment.

The multiple imputation approach described in section 4.4.6. will be applied to the baseline survey dataset to generate estimates of baseline prevalence or mean outcomes, as appropriate.

- - 1. **Exploratory analyses**

*Subgroup analyses – individual characteristics*

In conducting subgroup analyses there is a risk of false positive results and a lack of power to detect effects, it is therefore essential to rationalise a minimal list of characteristics to define the subgroups and to interpret the results with caution. The subgroup analyses will focus only on the primary trial outcomes.

The Cochrane Health Equity Field have made recommendations for assessing the impact of an intervention on health equity using subgroup analyses[[27](#_ENREF_27)]. These recommendations were developed for systematic reviews, however they are also relevant and informative for primary studies. The PROGRESS framework defines population subgroups that can be considered in subgroup analyses (PROGRESS: place of residence, race/ethnicity, occupation, gender, religion, education, socioeconomic status, social capital, age, disability, immigrant status). Subgroup analyses will be performed to investigate whether the Well London intervention had differential effects by:

Adults

- - - Age
    - Gender
    - Ethnicity
    - Level of education achieved (primary, GCSE, A-level, graduate)

Adolescents

- - - Age
    - Gender
    - Ethnicity
    - Family affluence scale score.

Stratified adjusted estimates will be produced using the methods described in section 4.4.4. separately for each subgroup.

Hypothesis tests will be conducted using the following steps for subgroups defined by binary variables (gender):

- - - 1. Calculate the difference in the outcome (mean or proportion) between the subgroups within each cluster
      2. Conduct a paired, LSOA-level t-test on the differences in outcome between the subgroups within each cluster.

Hypothesis tests for subgroups defined by ordinal categorical variables (age, education, social class) will be conducted by treating the categorical variable as a continuous variable and:

1. Fitting a regression slope of the categorical variable on the outcome
2. Comparing the slope coefficient between the pairs of LSOAs using a t-test.

*Subgroup analyses – neighbourhood characteristics*

The neighbourhood environment is a further potential source of modification of the effects of the Well London intervention on the primary outcomes. A cluster-level analysis will be used to determine if there is evidence of differential effects of Well London on:

- - - Physical activity by area of green space in the LSOA; walkability of the LSOA; cyclability of the LSOA; walking distance to green spaces of 2 hectares or larger; proximity to nearest sport or leisure facility;
    - Healthy eating by the number of shops selling fruit and vegetables; the number of fast food restaurants; proximity to nearest supermarket or food store; number of fast food restaurants at baseline;
    - All outcomes by resident turnover.

The measures of the neighbourhood characteristics are shown in Table 7. Many of these measures may have been modified during the 3.5 years of Well London programme delivery, particularly provision for cycling/cyclists and pedestrians, number of fast food restaurants and shops selling fruit and vegetables. Whilst we will use the baseline measures as moderator variables, we will examine changes in these characteristics over time by comparison between baseline and follow-up and consider the impact of any changes on the interpretation of the results.

The pair-matching will be relaxed for this subgroup analysis. The cluster-level outcome summaries (prevalence or mean scores), adjusted for key sociodemographic variables (as per section 4.4.4.) will be the dependent variable in an ANOVA with a categorical neighbourhood characteristic as the primary explanatory variable, and adjustment for treatment group (intervention/control status) and with an interaction between the neighbourhood variable and the treatment group. For neighbourhood characteristics represented by continuous variables, linear regression will be used with the same adjustment and interaction modelled.

Table 7: Measures of neighbourhood characteristics

| Measure | Description | Source | Source last updated |
| --- | --- | --- | --- |
| Resident turnover | Median duration of residence in the LSOA  % of residents migrating into LSOA since the start of Well London  % of residents migrating into LSOA since the final year of Well London  % of residents migrating into LSOA since Well London ended | Adult household survey follow-up (duration of residence in the LSOA) | N/a |
| Proximity to large parks/green spaces (2 hectares or larger) | Distance in kilometres to nearest park or green space | UK Department for Transport | 2009 |
| Proximity to sports/leisure facility | Distance in kilometres to nearest sports/leisure facility | Active Places Power (UK government planning tool) <http://www.activeplacespower.com/> | Continuous update |
| Area of green space in the LSOA | Meters^2^ of green space in the LSOA | Neighbourhood Statistics (UK government) <http://www.neighbourhood.statistics.gov.uk> | 2008 |
| Cycling provision | Density (per km^2^) of surveyed segments with a cycle lane (continuous or broken)  Density (per km^2^) of surveyed segments with traffic calming measures | Environmental audit baseline | N/a |
| Pedestrian provision | Density (per km^2^) of surveyed segments with traffic calming measures  Density (per km^2^) of surveyed segments with road-crossing aids | Environmental audit baseline | N/a |
| Number of shops selling fruit and vegetables at baseline | Density (per km^2^) of shops across the LSOA | Environmental audit baseline | N/a |
| Proximity to large supermarket/food store | Distance in kilometres to nearest food store or supermarket | UK Department for Transport | 2009 |
| Number of fast food restaurants | Density (per km^2^) of fast food restaurants across the LSOA | Environmental audit baseline | N/a |

*Mediation analyses*

The mediating effects of:

- - - adult residents’ perceptions of neighbourhood quality and safety and community cohesion on the primary adult outcomes;
    - the presence and intensity of signs of incivilities and social disorder at follow-up on the primary outcomes in adults;
    - mental wellbeing on changes in physical activity and healthy eating (adults and adolescents);
    - social capital on primary adolescent outcomes (Multidimensional Scale of Perceived Social Support);

will be examined by including these potential mediating variables as individual-level covariates using the method described for covariate adjustment in section 4.4.4. Substantial changes in the effect estimates after adjustment will be considered to indicate that these variables may be on the causal pathway between the Well London intervention and the primary outcomes. However, changes in effect estimates would also be seen if these covariates are in fact confounders of the relationship between the Well London intervention and the primary outcomes, therefore the logic model (see section 3.2.), and directed acyclic graphs based on the model, will be used to inform discussion of the results of these exploratory analyses.

Where appropriate methods using formal hypothesis tests to examine mediation effects will be used[[28-30](#_ENREF_28)].

*Exposure analyses*

Analysis of the exposure measures will address the following questions:

1. Do individuals who report direct participation in Well London activities have different outcomes to residents in the intervention LSOAs who do not report any participation? Is there evidence of a dose-response effect for the number of activities in which individuals have participated?
2. Do LSOAs with higher levels of self-report individual-level participation and more intense intervention delivery display greater community-level changes in the primary outcomes?

Evidence of the impact of direct exposure to the Well London intervention on the primary outcomes at the individual-level will be examined using multilevel regression of individual-level data from only the intervention LSOAs. This is analogous to an observational study of intervention exposure in the intervention areas only. The total levels of self-report participation will determine the method used: if there are small numbers of self-reported participants in each area a random effects model may be more appropriate, otherwise a fixed effects regression model with an indicator for LSOA will be used.

The impact of the community-level intensity of exposure to Well London on the primary outcomes will be investigated using a cluster-level analysis of the 20 intervention LSOAs. The method used will depend on the degree of between cluster variation in the outcomes before any covariate adjustments. If there is little between cluster variation a cluster-level regression analysis will be used, otherwise, a 2-stage process will be used to adjust for covariates (as per section 4.4.4.) and then perform a cluster-level regression. Four measures of community-level exposure will be used, with separate analyses for each:

- - - the number of quarters of project activity on offer (see Section 2.6 and Figure 1)
    - inclusion in phase 1 vs. phase 2 of delivery
    - the programme quarter in which the Well London Delivery Team was recruited, trained and became active
    - the level of self-report adult and adolescent participation in Well London activities.
    1. **Multiple Imputation**

The use of CAPI for the adult follow-up survey will substantially reduce the amount of missing responses for the adult outcomes. The expected levels of missing data for the adult follow-up survey are shown in Table 5 and are substantially lower than in the baseline survey for the primary outcomes and the majority of key socio-demographic and socio-economic characteristics. However, it may still be necessary to use multiple imputation for the secondary outcomes if the levels of missing data are higher, because these have not yet been assessed for the follow-up survey.

However the level of missing data in the adolescent outcomes from the follow-up survey is likely to be similar to that in the baseline survey (see Figure 1 to Figure 5). It will therefore be necessary to use multiple imputation to increase the efficiency of the adolescent outcome analyses and to reduce the impact of response bias.

*Imputation models*

For each variable in the outcome analysis models with any missing data, an imputation prediction model must be specified that is used to predict a value for those that are missing. This section describes the variables that will be used in the imputation prediction models for each of the outcomes and covariates described in sections 4.4.4 and 4.4.5.

The multiple imputation will be carried out separately for the intervention and control LSOAs. This is necessary to allow the relationship between the outcomes and the variables used to define the subgroups to vary between the intervention and control groups i.e. allowing for potential interactions between the subgroup variables and the intervention.

The Well London adult and adolescent survey datasets contain a large number of variables and only those variables which *a priori* are thought to plausibly hold substantial information about the outcomes or key covariates will be used in the imputation model. Any variables that will be explored as potential mediators or effect modifiers will also be included in the imputation models for the outcomes and vice versa.

For a given outcome (healthy eating, physical activity, mental wellbeing), each questionnaire item will be imputed separately and the overall composite outcome score calculated from these imputed items. For example, the GHQ12 adult mental health outcome has 12 questions from which the overall outcome score is calculated; missing responses for each of the 12 GHQ questions will be imputed then the overall score calculated from these imputed values. Table 8 and Table 9 show the variables used to impute each of the primary outcome composite score items and examples of the imputation models for the secondary outcomes and the sociodemographic and socioeconomic covariates.

An indicator for LSOA is included in the imputation to account for the clustering in the data, described in Section 4.2. There are no established computer algorithms for multilevel/random effects imputation models for binary categorical outcomes, therefore a fixed effects multiple imputation model in the current version of *ice* for Stata will be used. Although the number of LSOAs is large, estimates of the fixed (LSOA) effects should nonetheless be consistent since the cluster sample size is reasonably large at approximately 100 respondents per LSOA.

*Intervention exposure*

In the intervention LSOAs only, self-report participation in Well London will be included in the imputation models.

Table 8: Imputation model variables for the adult survey analyses

| Variable | Imputation model variables | |
| --- | --- | --- |
| Primary outcomes |  |  |
| Daily fruit and vegetable portion score composite item (n=11) | Other 10 fruit and vegetable portion score composite items  IPAQ score  WEMWBS score  GHQ12 score  Takeaway/fast food consumption  Fried food consumption  Sweet/savoury snacks consumption  Sugar sweetened beverage consumption  Diabetes  Heart condition  Self-report waist circumference, BMI  Desire to eat more healthily  Social networks  Social support  Social integration  Collective efficacy  Volunteering  Antisocial behaviour  Fear of crime | Age  Gender  Ethnicity  Level of education achieved  Marital status  Housing tenure  Ease of managing on household income  Smoking status  Alcohol consumption  Primary healthcare consultations in previous 12 months  Self-reported Well London participation (intervention LSOAs only)  LSOA |
| IPAQ score composite item (n=7) | Other 6 IPAQ score composite items  Daily fruit and vegetable portions  WEMWBS score  GHQ12 score  Takeaway/fast food consumption  Fried food consumption  Sweet/savoury snacks consumption  Sugar sweetened beverage consumption  Respiratory/breathing problems  Mobility problems  Diabetes  Heart condition  Self-report waist circumference, BMI  Desire to do more physical activity  Social networks  Social support  Social integration  Collective efficacy  Volunteering  Antisocial behaviour  Fear of crime | Age  Gender  Ethnicity  Level of education achieved  Marital status  Housing tenure  Ease of managing on household income  Smoking status  Alcohol consumption  Primary healthcare consultations in previous 12 months  Self-reported Well London participation (intervention LSOAs only)  LSOA |
| WEMWBS composite item (n=15) | Other 14 WEMWBS score composite items  Daily fruit and vegetable portions  IPAQ score  GHQ12 score  Hope scale score  Feeling anxious or depressed at time of interview  Consultation with a GP for mental or emotional problems in previous 12 months  Social networks  Social support  Social integration  Collective efficacy  Volunteering  Antisocial behaviour  Fear of crime | Age  Gender  Ethnicity  Level of education achieved  Marital status  Housing tenure  Ease of managing on household income  Smoking status  Alcohol consumption  Primary healthcare consultations in previous 12 months  Self-reported Well London participation (intervention LSOAs only)  LSOA |
| GHQ12 score composite item (n=12) | Other 11 GHQ12 score composite items  Daily fruit and vegetable portions  IPAQ score  WEMWBS score  Hope scale score  Feeling anxious or depressed at time of interview  Consultation with a GP for mental or emotional problems in previous 12 months  Social networks  Social support  Social integration  Collective efficacy  Volunteering  Antisocial behaviour  Fear of crime | Age  Gender  Ethnicity  Level of education achieved  Marital status  Housing tenure  Ease of managing on household income  Smoking status  Alcohol consumption  Primary healthcare consultations in previous 12 months  Self-reported Well London participation (intervention LSOAs only)  LSOA |
| Secondary outcomes – health behaviours – fried foods shown as an example |  |  |
| Consumption of fried foods; | Consumption of savoury snacks  Consumption of cakes and puddings  Consumption of sweets and chocolates  Consumption of sugar-sweetened soft drinks  Daily fruit and vegetable portions  IPAQ score  WEMWBS score  GHQ12 score  Diabetes  Heart condition  Self-report waist circumference, BMI  Desire to eat more healthily | Age  Gender  Ethnicity  Level of education achieved  Marital status  Housing tenure  Ease of managing on household income  Smoking status  Alcohol consumption  Primary healthcare consultations in previous 12 months  Self-reported Well London participation (intervention LSOAs only)  LSOA |
| Secondary outcomes – social & community processes – social networks shown as an example |  |  |
| Social networks - Contact with friends and neighbours | Social support  Social integration  Collective efficacy  Volunteering  Antisocial behaviour  Fear of crime  Daily fruit and vegetable portions  IPAQ score  WEMWBS score  GHQ12 score | Age  Gender  Ethnicity  Level of education achieved  Marital status  Housing tenure  Ease of managing on household income  Smoking status  Alcohol consumption  Primary healthcare consultations in previous 12 months  Self-reported Well London participation (intervention LSOAs only)  LSOA |
| Sociodemographic & socioeconomic characteristics – Age shown as an example |  |  |
| Age | Daily fruit and vegetable portions  IPAQ score  WEMWBS score  GHQ12 score  Takeaway/fast food consumption  Fried food consumption  Sweet/savoury snacks consumption  Sugar sweetened beverage consumption  Social networks  Social support  Social integration  Collective efficacy  Volunteering  Antisocial behaviour  Fear of crime | Gender  Ethnicity  Level of education achieved  Marital status  Housing tenure  Ease of managing on household income  Smoking status  Alcohol consumption  Primary healthcare consultations in previous 12 months  Self-reported Well London participation (intervention LSOAs only)  LSOA |
|  |  |  |

Table 9: Imputation model variables for the adolescent survey analyses

| Variable | Imputation model variables | |
| --- | --- | --- |
| Primary outcomes |  |  |
| PAQ-A score composite item (n=13) | Other 12 PAQ-A composite items  SDQ score  PANAS positive score  PANAS negative score  Portions of fruit and vegetables  MSPSS | School year  Gender  Ethnicity  LSOA |
| SDQ score composite item (n=25) | Other 24 SDQ composite items  PAQ-A score  PANAS positive score  PANAS negative score  MSPSS | School year  Gender  Ethnicity  LSOA |
| PANAS score composite item (n=20) | Other 19 PANAS score composite items  PAQ-A score  SDQ score  Portions of fruit and vegetables  MSPSS | School year  Gender  Ethnicity  LSOA |
| Portions of fruit and vegetables per day | PAQ-A score  SDQ score  PANAS positive score  PANAS negative score  Portions of fruit and vegetables  MSPSS | School year  Gender  Ethnicity  LSOA |
| Mediator variables |  |  |
| MSPSS composite item (n=12 items) | Other 11 MSPSS composite items  PAQ-A score  SDQ score  PANAS positive score  PANAS negative score  Portions of fruit and vegetables | School year  Gender  Ethnicity  LSOA |
| Socio-demographics |  |  |
| Gender | PAQ-A score  SDQ score  PANAS positive score  PANAS negative score  Portions of fruit and vegetables  MSPSS | School year  Ethnicity  LSOA |
| Ethnicity | PAQ-A score  SDQ score  PANAS positive score  PANAS negative score  MSPSS | School year  Gender  LSOA |
|  |  |  |

1. Scotland's Housing And Regeneration Project (2002-2008) [↑](#footnote-ref-1)
2. Research with East London Adolescents: Community Health Survey [↑](#footnote-ref-2)
3. The Schools and Students Health Education Unit, [↑](#footnote-ref-3)
4. The CMO recommendations for England between April 2004 and July 2011 were to accumulate at least 30 minutes of moderate intensity physical activity across five separate days (or the metabolic equivalent) for maintaining health. In July 2011 a joint statement from the CMOs of England, Wales, Scotland and Northern Ireland recommended that the 5x30mins per week target be retained but that accumulating a total of 150 minutes across the week (in bouts of at least 10 minutes) was likely to have similar benefits i.e. that 3x50mins would also be sufficient for maintaining health. [↑](#footnote-ref-4)
5. METs are multiples of the resting metabolic rate (1 MET is the resting metabolic rate, 2 METs are twice the resting metabolic rate) and indicate the intensity of a particular activity in terms of energy used; a MET-minute is computed by multiplying the MET score of a particular activity (defined in metabolic activity studies) by the minutes performed. [↑](#footnote-ref-5)
