# Supplementary material for: The Well London program - a cluster randomized trial of community engagement for improving health behaviors and mental wellbeing: baseline survey results
Source: Trials. 2012 Jul 6;13:105. doi: 10.1186/1745-6215-13-105 (PMC3441284; doi:10.1186/1745-6215-13-105)
Supplement: Additional file 3 — Missing data in regression variables from the Well London adult baseline survey. [file 1745-6215-13-105-S3.docx]

***Missing data in regression variables from the Well London adult baseline survey***

| Variable | Percent responses missing |
| --- | --- |
| Age | 9.2 |
| Gender | 1.8 |
| Ethnicity | 2.5 |
| Marital status | 2.7 |
| Employment status | 6.7 |
| Educational achievement | 11.1 |
| Housing tenure | 6.1 |
| Duration of residence in the LSOA | 6.8 |
| Ease of managing on household income | 6.8 |
| Healthy eating (portions of fruit and vegetables per day) | 11.9 |
| Physical activity (MET minutes per week) | 14.1 |
| Mental health |  |
| Hope scale | 11.0 |
| Self-report anxious or depressed (EQ5D) | 2.7 |
| Consult GP for anxiety/ depression/ emotional or nervous problem | 3.9 |

***Missing data in regression variables from the Well London adolescent baseline survey***

| Variable | Percent responses missing |
| --- | --- |
| School year | complete |
| Gender | 0.3 |
| Ethnicity | 3.3 |
| Duration of residence in the UK | 2.6 |
| Family Affluence Scale  Family owns a vehicle  Own bedroom at home  Family owns a computer  Family holidays this year | 1.5  0.4  0.7  4.9 |
| Healthy eating |  |
| Eat fruit daily or almost daily | 4.9 |
| Unhealthy eating score | 6.9 |
| Physical activity (PAQ-A score) | 27.0 |
| Mental health |  |
| PANAS positive | 20.8 |
| PANAS negative | 21.4 |
| SDQ | 18.9 |
