# Supplementary material for: The Well London program - a cluster randomized trial of community engagement for improving health behaviors and mental wellbeing: baseline survey results
Source: Trials. 2012 Jul 6;13:105. doi: 10.1186/1745-6215-13-105 (PMC3441284; doi:10.1186/1745-6215-13-105)
Supplement: Additional file 4 — Adult health behaviors and health outcomes; prevalences and means across all respondents, adjusted for clustering within LSOAs; based on the complete case dataset. [file 1745-6215-13-105-S4.docx]

***Adult health behaviours and health outcomes; prevalences and means across all respondents, adjusted for clustering within LSOAs; based on the complete case dataset.***

|  | K across all LSOAs | K_m_ across all LSOAs | ICC (р) | Sample size | | |
| --- | --- | --- | --- | --- | --- | --- |
| *Trial outcomes* |  |  |  | Intervention | Control | Total |
| Healthy eating – meeting five-a-day % | 0.20 | 0.14 | 0.03 | 1932 | 1904 | 3836 |
| Physical activity – meeting 5x30mins per week %  meeting 7x60mins per week % | 0.20  46 | 0.15  0.34 | 0.06  0.08 | 1746 | 1783 | 3529 |
| Mental health - mean Hope Scale score ^a^ | 0.04 | 0.02 | 0.05 | 1850 | 1807 | 3657 |
| Mental health - self-report feeling anxious or depressed % | 0.50 | 0.29 | 0.05 | 1932 | 1904 | 3836 |
| Mental health - self-report visit to general practitioner for anxiety/depression % | 0.72 | 0.23 | 0.10 | 2061 | 2046 | 4107 |

Abbreviations: CI, confidence interval; LSOA, lower super output area; ICC, intra-cluster correlation coefficient.

^a^ Higher score indicates greater hopefulness; maximum score 6 (delivered using 6-point likert scale responses).

***Adolescent health behaviours and health outcomes prevalences and means across all respondents, adjusted for clustering within LSOAs; based on the complete case dataset.***

|  | K across all LSOAs | K_m_ across all LSOAs | ICC (р) | Sample size | | |
| --- | --- | --- | --- | --- | --- | --- |
| *Trial outcomes* |  |  |  | Intervention | Control | Total |
| Diet  Eat fruit daily or almost daily %  Unhealthy eating – mean score^b^ | 0  0.05 | 0.004  0.06 | 0  0.01 | 585  569 | 583  573 | 1168  S1142 |
| Physical activity – mean PAQ score | 0.06 | 0.04 | 0.03 | 453 | 446 | 899 |
| Mental health – mean PANAS positive score  mean PANAS negative score | 0.03  0.02 | 0.02  0.02 | 0.03  0.01 | 494  493 | 479  475 | 973  968 |
| Mental health – mean SDQ score ^a^  normal SDQ score % | 0.02  0.10 | 0.02  0.05 | 0  0 | 490 | 506 | 996 |

Abbreviations: CI, confidence interval; LSOA, lower super output area; ICC, intra-cluster correlation coefficient.

^a^ Borderline score = 16-19; abnormal score>=20

^b^ Possible range 1-5; higher score indicates more frequent consumption of unhealthy food items (chips, chocolate or sweets, and sugar sweetened beverages
